# Multi-omics profiling identifies an immunometabolic signature associated with endometriosis

**DOI:** 10.3389/fendo.2026.1912463

**Published:** 2026-08-11

**Authors:** Wenwei Pan, Guizhen Lyu, Yikang Wang, Tingting Chen, Sainan Liu, Xinyi Fan, Ruibin Liu, Haochao Lin, Guanxia Li, Xiaofeng Su, Ping Zhou

**Affiliations:** 1 Department of Gynecology, Dongguan Hospital Affiliated To Shenzhen University, Dongguan, Guangdong, China; 2 Dongguan Key Laboratory of Female Reproductive Health, Dongguan, Guangdong, China; 3 Dongguan Labway Clinical Laboratory Co., Ltd., Dongguan, Guangdong, China; 4 The First School of Clinical Medicine, Guangdong Medical University, Zhanjiang, Guangdong, China; 5 Shanghai Labway Clinical Laboratory Co., Ltd., Shanghai, China

**Keywords:** endometriosis, immunometabolic reprogramming, metabolomics, multi-omics, proteomics

## Abstract

**Background:**

Endometriosis (EM) is a chronic inflammatory gynaecological disorder affecting approximately 10% of women of reproductive age. Early diagnosis remains challenging because definitive diagnosis still relies on invasive surgical confirmation. This study aimed to characterize systemic immune-metabolic alterations in EM and identify candidate peripheral blood biomarkers through integrated multi-omics profiling.

**Methods:**

Peripheral blood samples were collected from 88 participants, including 44 patients with EM T, 22 patients with benign ovarian cysts, and 22 healthy controls. Inflammatory proteomic profiling was performed using the Olink Target 96 Inflammation panel, and untargeted metabolomic analysis was conducted using ultra-high-performance liquid chromatography-high-resolution mass spectrometry (UHPLC-HRMS). Differential expression, pathway enrichment, integrative network analysis, and receiver operating characteristic (ROC) analyses with bootstrap resampling and false discovery rate (FDR) correction were performed.

**Results:**

Proteomic analysis revealed significant dysregulation of immune-inflammatory pathways in EM, characterized by enhanced chemokine signaling, cytokine-receptor interactions, and apoptotic activation. Metabolomic profiling identified substantial disturbances in energy metabolism, particularly involving fatty acid β-oxidation, acylcarnitine transport, and amino acid-carbon metabolic reprogramming. Integrated network analysis revealed a candidate immune-metabolic network comprising FGF21, CCL23, CDCP1, and CD8A. Correlation analysis demonstrated that FGF21 was positively associated with lipid oxidation-related metabolites, whereas CCL23 correlated with acylcarnitine species, indicating coordinated immune-metabolic interactions. ROC analyses showed that selected metabolite markers demonstrated moderate discriminatory performance between EM and healthy controls, while combined proteomic-metabolomic models achieved superior diagnostic performance compared with individual markers.

**Conclusion:**

Multi-omics profiling revealed coordinated immunometabolic alterations associated with EM, characterized by interconnected inflammatory and metabolic alterations in peripheral blood. These findings provide evidence that systemic immune-metabolic dysregulation is associated with EM and support further evaluation of blood-based biomarker strategies for non-invasive detection. However, these findings are based on a single-center cohort and require validation in independent populations and targeted assays.

## 1 Introduction

Endometriosis is a chronic gynecological disorder characterized by the ectopic implantation and growth of endometrial-like tissue outside the uterine cavity, and it increasingly shares biological characteristics with chronic low-grade inflammatory disorders, including systemic immunometabolic dysregulation, altered energy metabolism, endocrine dependence, and lifestyle-sensitive inflammatory burden (1, 2). The disease is associated with chronic inflammation, fibrosis, aberrant angiogenesis, and progressive tissue remodeling, resulting in a broad spectrum of clinical manifestations, including chronic pelvic pain, dysmenorrhea, dyspareunia, and infertility (3, 4). EM affects approximately 10% of women of reproductive age worldwide and more than 30% of women experiencing infertility (1, 5). Despite its substantial clinical burden, diagnosis remains challenging because early symptoms are often non-specific and definitive diagnosis still relies on invasive surgical visualization and histopathological confirmation (6). Commonly used biomarkers, including CA125, exhibit limited sensitivity and specificity, particularly in early-stage disease, restricting their utility for screening and disease monitoring (7). Consequently, the identification of accurate, minimally invasive biomarkers remains a major unmet clinical need in the management of EM. Advances in high-throughput omics technologies have transformed the investigation of complex diseases by enabling comprehensive characterization of molecular alterations across multiple biological layers. Proteomics provides insight into disease-associated changes in inflammatory signaling, immune regulation, and cellular communication networks (8), In contrast, metabolomics captures dynamic alterations in metabolic pathways, energy homeostasis, oxidative stress, and cellular bioenergetics (9, 10). Although individual omics platforms have generated valuable insights into EM pathogenesis, studies based on a single molecular layer often fail to capture the complex interactions between immune and metabolic processes that underlie disease progression (11). Multi-omics integration enables the systematic interrogation of interconnected molecular networks and has emerged as a powerful strategy for identifying biologically relevant pathways and candidate biomarkers in complex disorders (12). Accumulating evidence indicates that immune-metabolic reprogramming is a central feature of EM pathogenesis (13). Patients with EM exhibit persistent immune activation characterized by altered macrophage polarization, aberrant T-cell responses, impaired immune surveillance, and dysregulated cytokine production (14). Simultaneously, metabolic abnormalities, including altered mitochondrial function, enhanced fatty acid oxidation, oxidative stress, and disrupted amino acid metabolism, contribute to lesion survival and progression (15, 16). These immune and metabolic alterations are increasingly recognized as interconnected processes rather than independent pathological events. Metabolites such as acylcarnitines, fatty acid intermediates, and redox-related molecules can directly influence immune-cell activation, cytokine production, and inflammatory signaling pathways, thereby linking cellular metabolism with immune regulation (17, 18). Understanding these interactions is therefore essential for elucidating the systemic pathophysiology of EM.

Peripheral blood represents an attractive source for biomarker discovery because it captures systemic immune and metabolic alterations while allowing minimally invasive and repeatable sampling. Previous studies have identified EM-associated molecular changes using transcriptomic, proteomic, or metabolomic approaches (19); however, most investigations have focused on ectopic lesions, eutopic endometrium, or single-omics datasets. Consequently, the systemic immunometabolic landscape of EM remains incompletely characterized, and integrated analyses combining circulating proteomic and metabolomic profiles remain limited. Whether coordinated immune-metabolic alterations in peripheral blood can reveal biologically meaningful disease-associated networks and candidate biomarkers for non-invasive assessment of EM has not been fully explored.

Therefore, the present study employed integrated inflammatory proteomics and untargeted metabolomics to systematically characterize peripheral blood molecular alterations associated with EM. We hypothesized that systemic immune and metabolic pathways are coordinately dysregulated in EMand that integration of proteomic and metabolomic profiles would reveal biologically meaningful immunometabolic networks and candidate circulating biomarkers. By constructing a systems-level molecular framework, this study aimed to identify an immunometabolic signature associated with EM, provide insight into mechanisms linking inflammation and metabolic reprogramming, and establish a foundation for future blood-based diagnostic and translational studies.

## 2 Materials and methods

### 2.1 Study population and ethical approval

This study included a total of 88 peripheral blood samples collected from women undergoing gynaecological surgery at our hospital. The study protocol was approved by the Institutional Ethics Committee of Dongguan Hospital Affiliated to Shenzhen University, Dongguan (approval number: 202560), and written informed consent was obtained from all participants in accordance with the Declaration of Helsinki. Based on postoperative pathological diagnosis and clinical history, participants were classified into three groups: EM group (n = 44), comprising patients with surgically and histopathologically confirmed endometriosis; the benign ovarian cyst group (Benign, n = 22), consisting of patients with pathologically confirmed benign serous ovarian cysts; and the healthy control group (Healthy, n = 22), consisting of women without pelvic lesions, endocrine disorders, and inflammatory conditions confirmed by routine gynaecological examination and ultrasonographic assessment. The inclusion criteria were as follows: age between 20 and 45 years, regular menstrual cycles, and no hormone therapy within the previous three months. Exclusion criteria included pregnancy, diabetes mellitus, autoimmune diseases, active infection, malignant tumors, or other severe systemic disorders. Clinical variables collected for all participants included age, body mass index (BMI), menstrual cycle phase at blood collection, infertility history, serum CA125 levels, pain severity score (visual analogue scale, VAS), revised American Society for Reproductive Medicine (rASRM) stage, lesion size, and prior hormone treatment history. Menstrual cycle phase at the time of blood collection was recorded for all participants. However, because of the clinical availability of participants undergoing surgery and the exploratory nature of this multi-omics study, blood collection was not restricted to a specific menstrual cycle phase. Participants receiving hormonal therapy, including oral contraceptives or other hormonal medications, within three months before enrollment were excluded to minimize treatment-related metabolic effects. Other medications were not systematically analyzed because of limited sample size and incomplete medication records. The EM patients were diagnosed based on surgical visualization and histopathological confirmation. Disease severity was assessed according to the revised American Society for Reproductive Medicine (rASRM) classification; however, due to the exploratory nature of this multi-omics study and the limited sample size, subgroup analysis stratified by disease stage was not performed. Given the exploratory nature of this multi-omics study, sample size was determined based on sample availability and feasibility. Preliminary power estimation indicated that the current cohort size was sufficient to detect moderate-to-large effect sizes for pairwise comparisons. For all participants, 3 mL of fasting peripheral blood was collected in the early morning 7 am. Serum samples were isolated by centrifugation and stored at −80 °C until subsequent proteomic and metabolomic analyses. Serum samples were selected because they provide a stable and widely used matrix for circulating metabolomic and inflammatory biomarker profiling. All samples were processed using a standardized fasting blood collection protocol and stored under identical conditions to minimize pre-analytical variability. Clinical variables, including BMI, CA125 levels, VAS score, rASRM stage, and menstrual cycle phase, were collected for baseline characterization. However, these variables were not incorporated into the primary multi-omics models because of the exploratory design and limited cohort size.

### 2.2 Inflammatory proteomic profiling

Inflammatory proteomic profiling was performed using the Olink^®^ Target 96 Inflammation panel (Olink Proteomics AB, Uppsala, Sweden), based on proximity extension assay (PEA) technology, to quantify 92 inflammation-related proteins in serum samples. Briefly, paired oligonucleotide-labeled antibodies bind target proteins in proximity, enabling DNA hybridization and extension, followed by quantitative real-time PCR amplification on the Olink Signature Q100 platform. Raw cycle threshold (Ct) data were processed using Olink NPX Manager software according to the manufacturer’s standard pipeline. Protein abundance was normalized and reported as Normalized Protein eXpression (NPX) values on a log2 scale, where higher NPX values indicate higher relative protein abundance. Quality control (QC) procedures were performed using built-in internal controls, including incubation, extension, and detection controls, together with external negative controls and inter-plate controls (IPCs), to monitor assay performance and technical variation. Run-level QC required a standard deviation <0.2 for extension and detection controls, while sample-level QC required NPX deviation within ±0.3 of the plate median. Samples were evaluated according to predefined QC criteria. All 88 samples passed quality control requirements and were retained for downstream analyses. All assays were performed in a single experimental batch to minimize inter-batch variation and ensure analytical consistency. Final NPX data were exported for downstream differential expression, correlation, and integrative multi-omics analyses.

### 2.3 Differential protein analysis and functional annotation

Normalized proteomic data generated by Olink NPX Manager were imported into R (v4.3.1) for downstream statistical analysis. Differential protein expression between groups was assessed using the limma package (v3.58.0), and log2 fold changes (log2FC) with corresponding p-values were calculated. Proteins with |log2FC| > 0.58 and adjusted p-values (q-values) < 0.05 were considered significantly differentially expressed. To reduce false-positive findings arising from high-dimensional omics analyses, multiple testing correction was performed using the Benjamini-Hochberg false discovery rate (FDR) method. Unless otherwise specified, FDR-adjusted q-values < 0.05 were considered statistically significant. Group-wise comparisons of representative protein expression levels were performed using appropriate statistical tests, and results were visualized using boxplots to display data distribution. Hierarchical clustering heatmaps of differential proteins were generated using the pheatmap package, with bidirectional clustering based on Euclidean distance and the Ward.D2 method. Protein-protein correlation analyses were performed using Pearson correlation coefficients. To investigate the biological significance of differential proteins, functional enrichment analyses were performed using the clusterProfiler package. Gene Ontology (GO), Kyoto Encyclopedia of Genes and Genomes (KEGG), and Reactome pathway enrichment analyses were conducted, and pathways with FDR-adjusted *p*-values < 0.05 were considered significantly enriched. Enrichment ratios (GeneRatio) were calculated to quantify pathway representation.

### 2.4 Untargeted metabolomic profiling

Serum samples were thawed at 4 °C and vortex-mixed thoroughly prior to processing. For metabolite extraction, 100 μL of serum was mixed with 100 μL methanol/acetonitrile (1:1, v/v), vortexed for 1 min, and sonicated in an ice bath for 10 min. Samples were centrifuged at 12,000 rpm for 10 min at 4 °C, and the supernatants were collected and vacuum-dried. The dried extracts were reconstituted in 150 μL of 80% methanol containing 4 ppm 2-chloro-L-phenylalanine as an internal standard and filtered through a 0.22 μm PTFE membrane before analysis. Quality control (QC) samples were prepared by pooling equal aliquots from all samples and injected every 20 runs to monitor instrument stability and analytical reproducibility. All sample preparation procedures were completed in a single batch to minimize technical variation. Untargeted metabolomic profiling was performed using an ultra-high-performance liquid chromatography-high-resolution mass spectrometry system (UHPLC-Q Exactive Orbitrap, Thermo Fisher Scientific, USA). Chromatographic separation was achieved using an ACQUITY UPLC HSS T3 column (2.1 × 100 mm, 1.8 μm, Waters, USA) maintained at 40 °C with a flow rate of 0.3 mL/min. Data acquisition was conducted in both positive and negative electrospray ionization (ESI) modes using a data-dependent acquisition (Full MS-ddMS2) workflow. Mass spectra were acquired over an m/z range of 100–1000 with an MS1 resolution of 70,000 and an MS/MS resolution of 17,500. Raw LC-MS data were processed using Compound Discoverer 3.1 (Thermo Fisher Scientific), including peak detection, retention time alignment, signal normalization, and feature quantification. Features with relative standard deviation (RSD) >30% in QC samples were excluded to improve analytical robustness. Metabolite annotation was performed by matching MS/MS spectra against the Human Metabolome Database (HMDB), KEGG, and Metlin databases, using a mass tolerance of <10 ppm and spectral similarity ≥80%. Metabolite annotation confidence was classified according to the Metabolomics Standards Initiative (MSI), with MS/MS spectral matching regarded as MSI level 2 and reference-standard confirmation as MSI level 1 where available. Normalized metabolite intensities were log2-transformed for downstream statistical and integrative analyses. Procedural blank samples were not included in the original experimental workflow. Therefore, the possibility of low-abundance environmental or reagent-derived contamination cannot be completely excluded. Candidate exogenous metabolites identified in this study should therefore be interpreted cautiously and require confirmation using targeted analytical methods with appropriate blank controls.

### 2.5 Multivariate analysis and differential metabolite selection

To characterize metabolic differences between groups, supervised multivariate analyses were performed using partial least squares discriminant analysis (PLS-DA) and orthogonal partial least squares discriminant analysis (OPLS-DA) implemented in the ropls package (v1.28.2) in R. Prior to modeling, normalized metabolite data were Pareto-scaled to reduce the influence of large-intensity variables while preserving data structure. Model performance and robustness were evaluated using seven-fold cross-validation based on R2Y and Q2 metrics, and potential overfitting was assessed by 100 permutation tests. Models were considered acceptable when showing high explanatory power (R2Y), predictive performance (Q2), and no evidence of overfitting in permutation analysis. Variable importance in projection (VIP) scores derived from the OPLS-DA model were used to rank discriminative metabolites. To improve statistical rigor and reduce false-positive findings, differential metabolite selection was based on combined multivariate and univariate criteria, including VIP >1, |log2FC| >0.58, and Benjamini-Hochberg FDR-adjusted q-values <0.05. The final set of significant metabolites was used for downstream pathway enrichment, protein-metabolite correlation analysis, and integrative multi-omics network construction.

### 2.6 Functional enrichment analysis

To systematically characterize the biological functions and signaling pathways associated with differential proteins and metabolites, enrichment analyses were performed using the clusterProfiler package (v4.10.0) in R (v4.3.1), the ReactomePA package, and the MetaboAnalyst 6.0 platform. For proteomic analysis, differential proteins were converted to Entrez gene IDs using the org.Hs.eg.db database (v3.16.0) and subjected to Gene Ontology (GO), Kyoto Encyclopedia of Genes and Genomes (KEGG), and Reactome pathway enrichment analyses. Pathways with Benjamini-Hochberg FDR-adjusted *p*-values <0.05 and containing at least three enriched genes were considered statistically significant. For metabolomic analysis, differential metabolites were annotated using standardized identifiers from the Human Metabolome Database (HMDB), KEGG, Lipid Maps, and SMPDB databases. Pathway enrichment and topology analyses were performed using the “Enrichment Analysis” and “Pathway Analysis” modules in MetaboAnalyst. Pathway enrichment significance was evaluated using both nominal *p*-values and Benjamini–Hochberg false discovery rate (FDR)-adjusted p-values. Pathways with FDR-adjusted *p*-values <0.05 and pathway impact values ≥0.1 were considered statistically significant. Nominal *p*-values are reported for descriptive interpretation of pathway ranking, whereas biological interpretation was based primarily on FDR-adjusted significance. To identify shared biological pathways across proteomic and metabolomic datasets, joint KEGG pathway enrichment analysis was performed by mapping differential proteins and metabolites to common KEGG pathways. Statistical significance of pathway overlap between omics layers was evaluated using Fisher’s exact test, enabling the identification of coordinated immune-metabolic pathways associated with EMT. Enrichment results were visualized using bubble plots and bar plots generated with ggplot2. Integrated protein-metabolite-pathway interaction networks were constructed and visualized in Cytoscape (v3.9.1) to illustrate system-level molecular interactions across omics layers.

### 2.7 Mantel correlation and system-level coupling analysis

To evaluate the degree of system-level coupling and overall coordination between the proteomic and metabolomic datasets, Mantel tests were first performed using standardized matrices of differential proteins and metabolites. Euclidean distance matrices were computed via the mantel() function in the vegan package (v2.6-6) in R (v4.3.1), followed by 9,999 permutations to obtain Mantel r statistics and associated p-values. The Mantel correlation coefficient (r > 0 indicates a positive association) quantified the structural similarity between the two omics layers, and a significance threshold of p < 0.05 was used to identify statistically significant correlations. To further explore pairwise linear associations between proteins and metabolites, Pearson correlation coefficients (r) were calculated using the Hmisc (v4.7.0) and psych (v2.2.9) packages. Correlated pairs were selected based on thresholds of |r| > 0.4 and *p* < 0.001. When a large number of significant associations were identified, the top 50 protein-metabolite pairs were selected for visualization. Heatmaps were generated using the pheatmap package, with rows and columns representing metabolites and proteins, respectively. Color gradients indicated the strength of the correlation (red for positive and blue for negative), and hierarchical clustering (method = “ward.D2”) was applied to both dimensions. To visually depict system-level regulatory patterns, protein-metabolite interaction networks were constructed using the ggraph (v2.1.0) and igraph (v1.6.0) packages. Node shapes distinguished molecular types—circles for metabolites and triangles for proteins—while node size represented degree centrality. Edge thickness and color corresponded to the strength of Pearson correlation. Topological parameters, including degree, betweenness, and closeness centrality, were computed using igraph to identify key hub molecules within the network. Final visualizations were optimized in Cytoscape (v3.9.1), with KEGG-based pathway layers integrated to generate a multidimensional map of protein-metabolite-pathway interactions. Mantel correlation analysis was used to evaluate global concordance between proteomic and metabolomic distance structures and was interpreted independently from pairwise Pearson correlations. Pearson correlation analysis was applied to identify individual protein–metabolite associations, whereas Mantel statistics reflected overall similarity between molecular datasets rather than direct molecular interaction strength.

### 2.8 Multi-omics network construction and identification of key nodes

To systematically elucidate the interactions between differential proteins and metabolites, as well as their central roles within the inflammation-metabolism regulatory network, a protein-metabolite interaction network was first constructed using the igraph package (v1.6.0) in R (v4.3.1), based on Mantel and Pearson correlation results (|r| > 0.4, *p* < 0.05). The multi-omics integration strategy was designed as a complementary framework combining correlation-based molecular network analysis, pathway-based biological interpretation, and model-based feature prioritization. Protein–metabolite relationships were first evaluated through correlation analyses and network construction, followed by joint pathway enrichment to identify shared biological processes. Machine learning-based prioritization and logistic regression modeling were subsequently applied to identify molecular features with potential discriminatory value. In this network, edges represented significant correlations between proteins and metabolites, with edge weights defined by the absolute value of the correlation coefficient (r). Nodes corresponded to molecular entities, with node size scaled by degree centrality and node color mapped to the weighted correlation coefficient (red for positive, blue for negative correlations). Edge thickness and transparency reflected the strength of the correlation. Subsequent visualization and topological analysis were performed in Cytoscape 3.9.1. Topological parameters—including degree, betweenness centrality, and closeness centrality—were calculated using the NetworkAnalyzer plugin. Betweenness centrality was used to assess a molecule’s role as a bridge in information flow. Hub nodes were defined as those ranking within the top 10% in betweenness centrality. To identify molecular features with the most significant contribution to the integrative omics structure, a random forest model was constructed using the randomForest package (v4.7-1.1) in R. The input features consisted of a matrix of differential proteins and metabolites, and the output classification labels were experimental versus control. Model parameters were set as ntree = 1000 and mtry = √ (number of variables). The Gini importance index (MeanDecreaseGini) was calculated for each variable, and the top 10 most important features were selected and visualized in a bar plot. Model performance was evaluated using 10-fold cross-validation to estimate internal performance and reduce overfitting risk. Finally, by integrating the results from the random forest model and network topology analysis, molecules with both high betweenness centrality (top 10%) and high Gini importance were identified as potential key regulatory nodes.

### 2.9 Diagnostic performance evaluation

To assess the discriminatory power of candidate molecules in the diagnosis of EM, receiver operating characteristic (ROC) curves were generated using the pROC package (v1.18.4) in R (v4.3.1), and the area under the curve (AUC), sensitivity, and specificity were calculated. For multi-marker combination analysis, a multivariate logistic regression model was constructed using the glm() function (family = binomial), and the combined predictive probability was calculated as logit(p) = β0 + β_1_X_1_ + β2X2 +… + βnXn. To prevent overfitting and optimize feature selection, stepwise regression in both directions was applied based on the minimum Akaike Information Criterion (AIC). The predictive performance of the models was evaluated using 10-fold cross-validation to estimate internal generalizability. Bootstrap resampling (n = 1000 iterations) was additionally performed to assess the stability and uncertainty of the ROC estimates. Bootstrap-derived AUC values were calculated as the mean AUC across resampled datasets and were interpreted as internal validation estimates rather than improvements over the original observed AUC. An AUC >0.80 was considered to indicate high discriminatory ability, 0.70–0.80 moderate discriminatory ability, and <0.70 limited discriminatory ability. Optimal cut-off values for sensitivity, specificity, and accuracy were determined using the Youden Index (J = Sensitivity + Specificity − 1). The predictive capacity of the combined model was further assessed by calibration analysis using the Hosmer-Lemeshow goodness-of-fit test (*p* > 0.05 indicating good fit) and the Nagelkerke R2 statistic. To visually illustrate the model’s discriminative performance, prediction probability distribution plots and calibration curves were generated. Confusion matrices were computed to evaluate positive predictive value (PPV) and negative predictive value (NPV). Ultimately, the most clinically promising biomarker combinations were identified based on ROC comparisons and regression coefficients (β values) from the logistic regression output. Spearman correlation analysis was performed to evaluate the associations between serum CA125 levels and key molecular features identified from the proteomic and metabolomic analyses. Correlation coefficients (r) and corresponding p-values were calculated, and multiple comparisons were corrected using the Benjamini–Hochberg false discovery rate (FDR) method. An adjusted p-value <0.05 was considered statistically significant.

### 2.10 Metabolite identification confidence and statistical robustness analysis

To address metabolite annotation confidence, all MS/MS-annotated metabolites were categorized according to the Metabolomics Standards Initiative (MSI) confidence framework using the annotation level reported by the metabolomics pipeline. Metabolites confirmed by reference-level spectral evidence were annotated as MSI level 1, metabolites supported by MS/MS spectral library matching were annotated as MSI level 2, and metabolites assigned at lower structural or class-level confidence were annotated as MSI level 3. The distribution of MSI levels was summarized for all identified metabolites and for candidate diagnostic metabolites. To improve the statistical robustness of diagnostic evaluation, ROC analyses were repeated for all quantified Olink proteins and MS/MS-annotated metabolites in each pairwise comparison. AUC values were calculated using the pROC package, and 95% confidence intervals for candidate markers were estimated by 2,000 stratified bootstrap resampling iterations. For feature-wise diagnostic screening, Wilcoxon rank-sum *p*-values were corrected using the Benjamini-Hochberg method across all 1,464 evaluated molecular features within each comparison. To estimate the internal robustness of candidate molecular panels identified from differential analysis and multi-omics prioritization, repeated stratified 5-fold cross-validation was performed 100 times. Logistic regression models were trained on standardized features in each training fold and evaluated on the held-out fold; cross-validated AUC values were summarized as the mean and standard deviation across repeats. To evaluate whether protein-metabolite associations were driven only by group separation, group-adjusted correlation analysis was performed as a sensitivity analysis. Each protein and log2-transformed metabolite feature was first residualized using a linear model with diagnostic group as the covariate. Spearman correlations were then calculated between residualized protein and metabolite values across paired samples, followed by Benjamini-Hochberg FDR correction across all protein-metabolite pairs. Candidate biomarker panels were defined based on prior differential abundance analysis, biological relevance, and multi-omics integration results before machine-learning modeling. Random Forest models were subsequently evaluated using 10-fold cross-validation and bootstrap resampling (n = 1000) to assess internal robustness and generalizability. Because this study was designed as an exploratory biomarker discovery analysis with a limited sample size, fully nested feature selection within each cross-validation fold was not performed. Therefore, model performance estimates should be considered internally validated and require confirmation in independent cohorts.

## 3 Results

### 3.1 Differential proteomic profiling identifies distinct circulating inflammatory signatures in endometriosis

**Figure 1:**
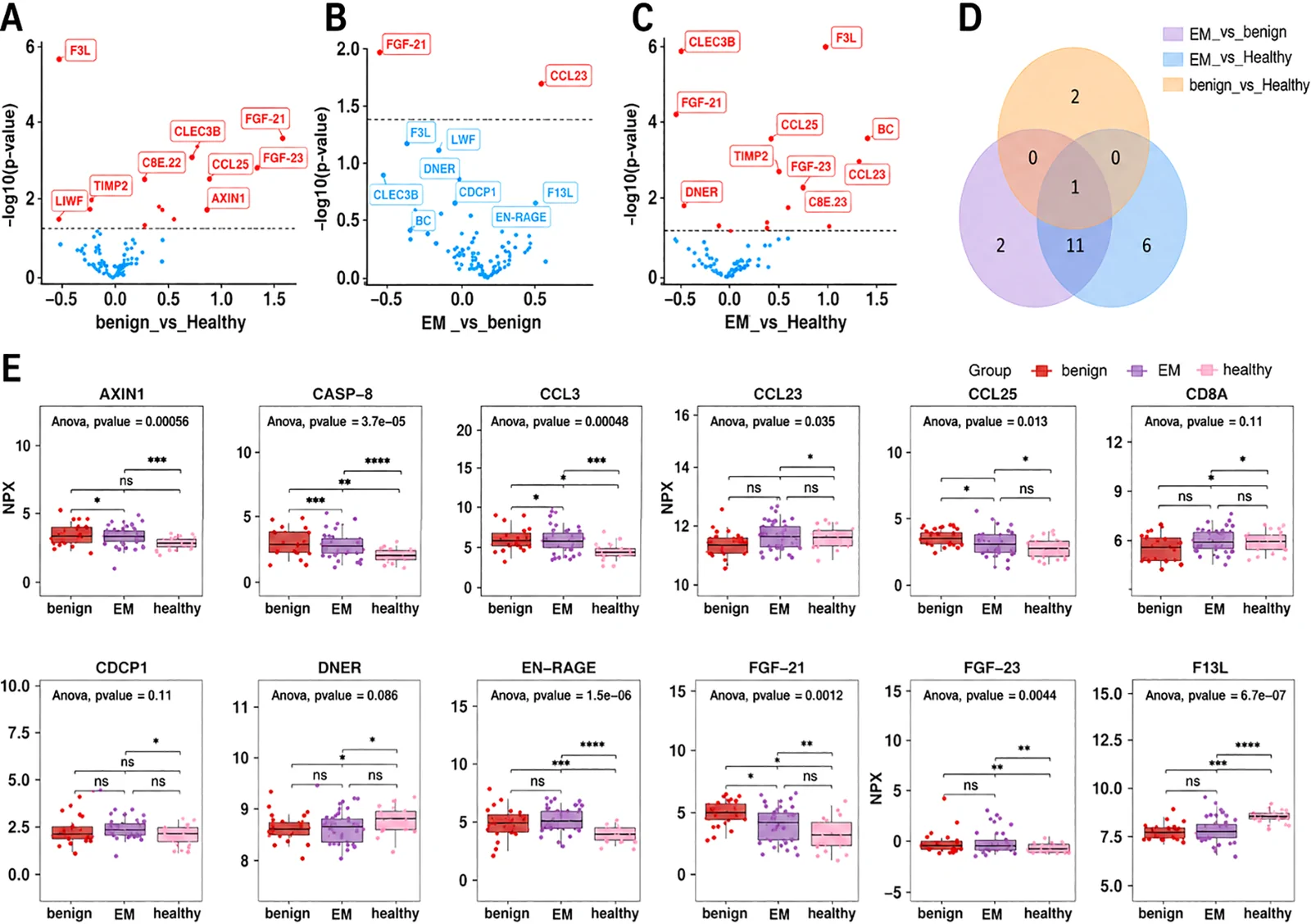
Differential proteomic profiling reveals immune-inflammatory alterations associated with endometriosis. **(A–C)** Volcano plots showing differentially expressed proteins in the Benign versus Healthy **(A)**, EM versus Benign **(B)**, and EM versus Healthy **(C)** comparisons. Red dots indicate significantly differentially expressed proteins, whereas blue dots indicate non-significant proteins. Selected proteins are annotated. **(D)** Venn diagram illustrating the overlap of differentially expressed proteins among pairwise comparisons. **(E)** Expression distributions of representative inflammatory and immune-related proteins across Healthy, Benign, and EM groups. Statistical significance was evaluated by one-way ANOVA followed by multiple-comparison testing. *P < 0.05, **P < 0.01, ***P < 0.001, ****P < 0.0001.

To characterize systemic inflammatory alterations associated with EM, serum proteomic profiling was performed using the Olink Target 96 Inflammation panel across 88 samples, including Benign (n = 22), EM (n = 44), and Healthy (n = 22) groups. The overall experimental workflow is shown in **Supplementary Figure 1A**. Quality control analysis demonstrated high assay stability and reproducibility. NPX distribution boxplots showed generally consistent signal distributions across all samples, with only a small number of samples flagged as QC warnings (**Supplementary Figure 1B**). Sample median–interquartile range (IQR) plots further confirmed that all samples fell within acceptable QC thresholds without evident outliers (**Supplementary Figure 1C**), supporting the robustness of the proteomic dataset for downstream analysis. Pairwise differential expression analysis was performed using normalized NPX values. Proteins meeting the predefined thresholds (|log2FC| > 0.58 and FDR-adjusted q-values < 0.05) were considered significantly differentially expressed and visualized by volcano plots (**Figures 1A–C**). In the Benign versus Healthy comparison, 17 proteins were differentially expressed, including Flt3L, FGF21, CASP8, CCL3, IL6, IL8, TWEAK, CCL25, AXIN1, and uPA (**Figure 1A**). In the EM versus Benign comparison, two proteins met the significance threshold, with CCL23 significantly upregulated and FGF21 significantly downregulated in the EM group (**Figure 1B**). In the EM versus Healthy comparison, 20 proteins were significantly altered, including Flt3L, CASP8, CCL3, IL6, IL8, EN-RAGE, FGF23, TWEAK, AXIN1, and MCP3 (**Figure 1C**). To assess the overlap of differential proteins across comparisons, Venn diagram analysis was performed (**Figure 1D**). Fourteen proteins overlapped between the Benign versus Healthy and EM versus Healthy groups, indicating shared inflammatory alterations across disease-associated states. To further evaluate the expression differences of representative candidate proteins among the three groups, statistical comparisons of protein abundance were performed using group-wise comparisons, with the corresponding distributions visualized by boxplots (**Figure 1E**). CCL23 showed significantly higher expression in the EM group compared with the Benign group (*p* = 0.035), whereas FGF21 was significantly lower in the EM group (*p* = 0.0012). CD8A showed a modest increase in EM relative to Benign, although the overall group-level variation was less pronounced. Additional proteins, including CASP8, CCL3, EN-RAGE, FGF23, and Flt3L, also demonstrated significant intergroup differences, supporting broad alterations in circulating inflammatory profiles. These findings demonstrate that EM is associated with distinct peripheral inflammatory protein alterations and identify candidate molecules for subsequent integrative proteomic-metabolomic analysis. In the EM versus Healthy comparison, FGF21 showed a similar directional trend but did not reach statistical significance, suggesting that the observed reduction may partially reflect differences between benign and EM groups.

### 3.2 Functional enrichment of differential proteins highlights chemokine and cytokine signaling in endometriosis

**Figure 2:**
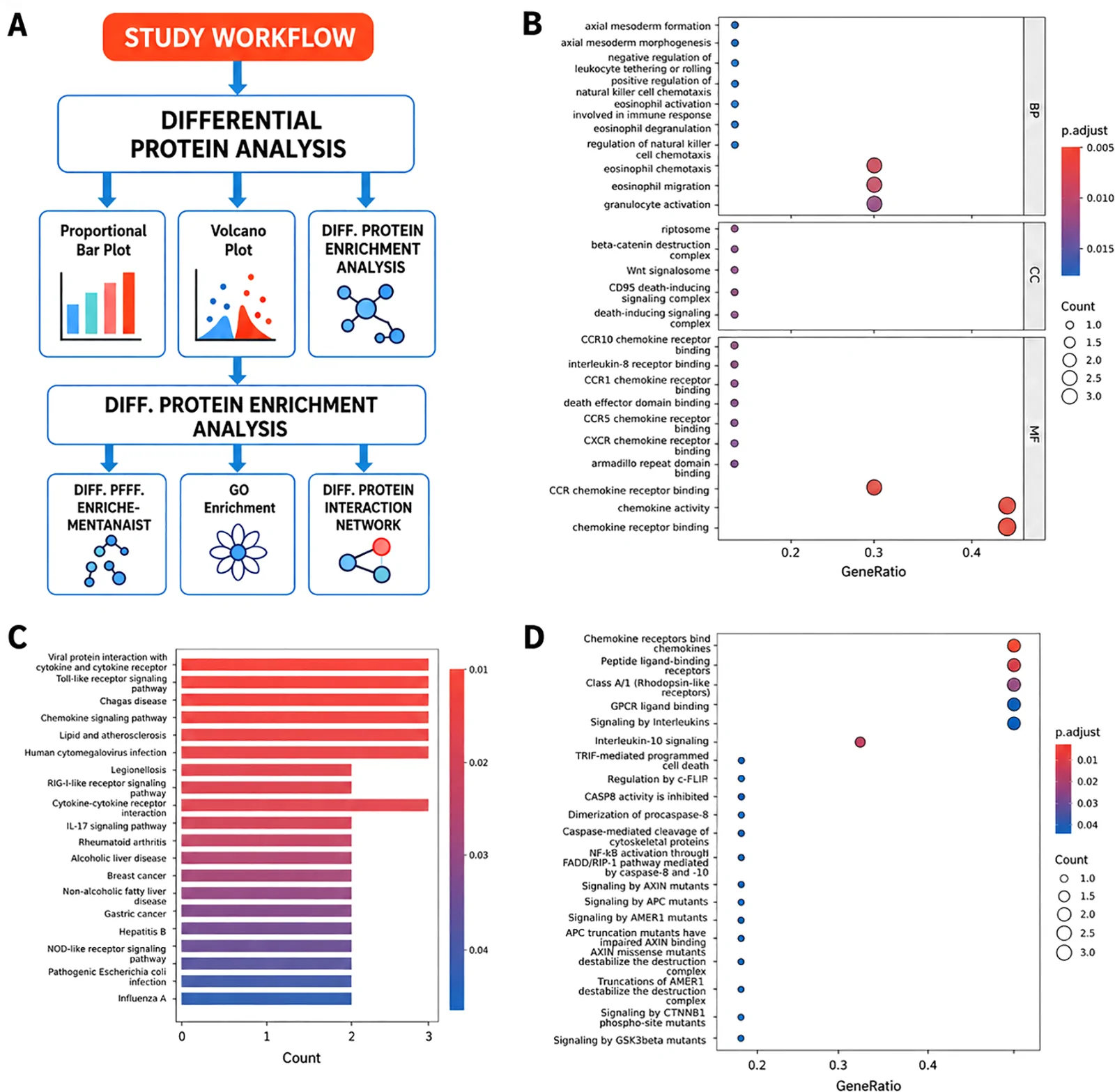
Functional enrichment analysis and pathway identification of differentially expressed proteins. **(A)** Schematic overview of the proteomic analysis workflow, including differential protein identification, enrichment analysis, and network-based functional characterization. **(B)** Gene Ontology (GO) enrichment analysis of differentially expressed proteins. Enriched terms are grouped into Biological Process (BP), Cellular Component (CC), and Molecular Function (MF) categories. Bubble size represents the number of proteins associated with each term, and color indicates the adjusted p-value. **(C)** Kyoto Encyclopedia of Genes and Genomes (KEGG) pathway enrichment analysis showing significantly enriched pathways among differential proteins. Bar color represents the adjusted p-value. **(D)** Reactome pathway enrichment analysis of differential proteins. Bubble size indicates the number of enriched proteins within each pathway, and color denotes the adjusted p-value.

To investigate the biological relevance of differentially expressed proteins, functional enrichment analyses were performed using GO, KEGG, and Reactome databases (**Figure 2A**). GO enrichment analysis showed that differential proteins were predominantly enriched in biological processes related to immune activation and leukocyte chemotaxis (**Figure 2B**). The most significantly enriched biological process terms included granulocyte activation, eosinophil migration, eosinophil degranulation, and natural killer cell chemotaxis. In the cellular component category, enriched terms included the beta-catenin destruction complex and CD95 death-inducing signaling complex. Molecular function analysis revealed strong enrichment for chemokine receptor binding, CCR chemokine receptor binding, and chemokine activity. KEGG pathway analysis demonstrated significant enrichment in inflammation-related signaling pathways (**Figure 2C**), including cytokine-cytokine receptor interaction, chemokine signaling pathway, Toll-like receptor signaling pathway, RIG-I-like receptor signaling pathway, and IL-17 signaling pathway. Additional enrichment in broader disease-associated pathways, such as lipid and atherosclerosis, likely reflected shared inflammatory signaling components rather than disease-specific mechanisms. Reactome pathway analysis further supported these findings, showing enrichment in chemokine receptor interactions, interleukin signaling, interleukin-10 signaling, TRIF-mediated programmed cell death, and caspase-related signaling modules (**Figure 2D**). Several apoptosis-related pathways, including caspase-8 activity and FADD/RIP1-mediated signaling, were also significantly represented. Present results enrichment analyses demonstrate that differential circulating proteins in EM are primarily associated with immune chemotaxis, cytokine signaling, and apoptosis-related pathways, supporting the presence of systemic inflammatory dysregulation in EM.

### 3.3 Random forest prioritization identifies candidate discriminatory proteins in endometriosis

**Figure 3:**
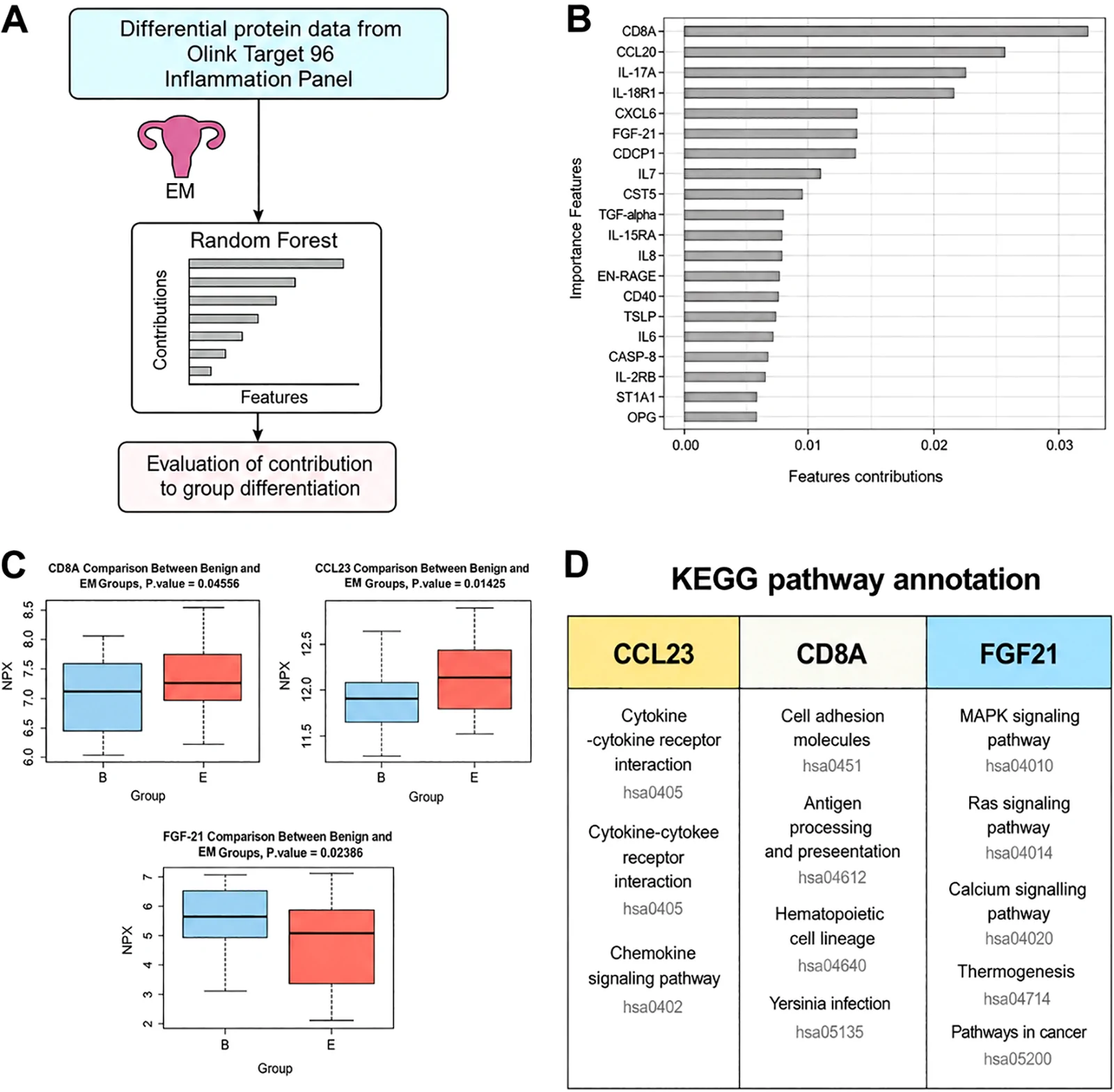
Identification and validation of key differential proteins distinguishing EMfrom benign based on the olink target 96 Inflammation Panel. **(A, B)** Random Forest analysis ranking the contribution of each differential protein to group classification; **(C)** Expression profiles of key proteins between the Benign and EM groups; **(D)** KEGG pathway annotation showing the functional pathways enriched by the key proteins.

To prioritize proteins with the greatest contribution to group discrimination, a random forest model was constructed using differential proteins derived from the Olink Target 96 Inflammation panel (**Figure 3A**). Feature importance analysis ranked proteins according to their relative contribution to distinguishing the EMand Benign groups (**Figure 3B**). Among the top-ranked proteins, CD8A showed the highest contribution score, followed by CCL20, IL-17A, IL-18R1, CXCL6, FGF21, and CDCP1. To further evaluate representative candidate proteins, differential expression analysis was performed between the EMand Benign groups (**Figure 3C**). CCL23 and CD8A showed significantly higher NPX values in the EMgroup, whereas FGF21 showed significantly lower expression. These consistent expression differences supported their prioritization for downstream integrative analysis. KEGG pathway annotation was performed to contextualize the potential biological relevance of these candidate proteins (**Figure 3D**). CCL23 was annotated to cytokine-cytokine receptor interaction and chemokine signaling pathways, CD8A was linked to antigen processing and presentation and hematopoietic cell lineage pathways, and FGF21 was associated with MAPK signaling, Ras signaling, calcium signaling, and thermogenesis-related pathways. These analyses identified CCL23, CD8A, and FGF21 as candidate discriminatory proteins associated with EMand prioritized them for subsequent integrative proteomic-metabolomic network analysis.

### 3.4 Untargeted metabolomics reveals distinct systemic metabolic alterations in endometriosis

**Figure 4:**
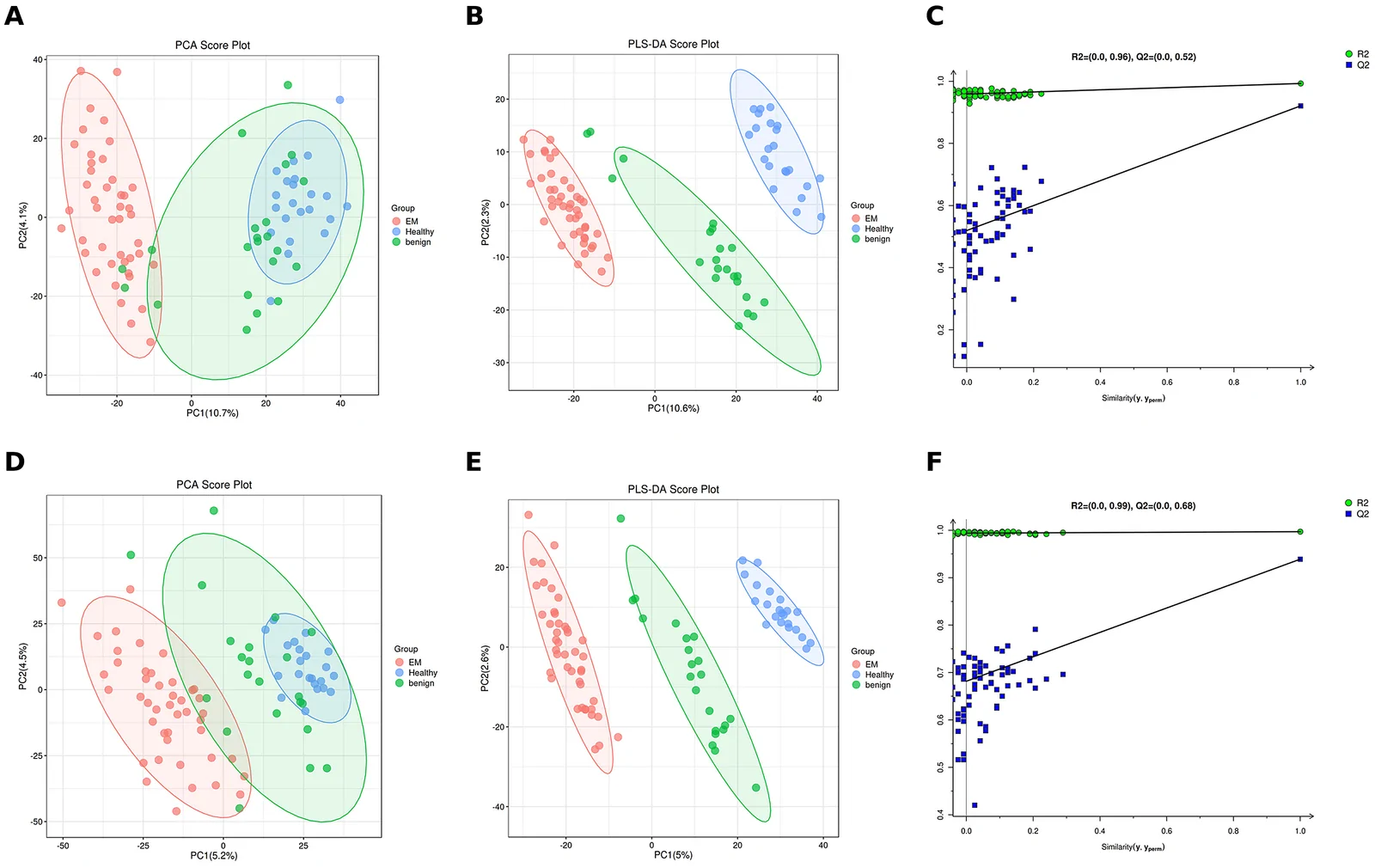
Multivariate statistical analysis of metabolomics data in positive and negative ion modes. **(A–C)** PCA and PLS-DA of negative ion mode data. PCA plots display the spatial distribution of samples across groups; PLS-DA modeling and validation indicate R2 = 0.96 and Q2 = 0.52; **(D–F)** PCA and PLS-DA of positive ion mode data, with model performance showing R2 = 0.99 and Q2 = 0.68. Sample groups (EMT, Benign, Healthy) are visualized within the metabolic profile space along with model validation metrics.

To further characterize systemic metabolic alterations associated with EM, untargeted metabolomic profiling was performed on serum samples from the EM, Benign, and Healthy groups. Quality control assessment demonstrated good analytical stability and reproducibility. Principal component analysis of QC samples showed tight clustering in both negative and positive ion modes (**Supplementary Figures 2A, C**), indicating stable instrument performance and minimal batch variation. Consistently, relative standard deviation (RSD) analysis showed that 70.0% of detected features in negative ion mode and 73.5% in positive ion mode exhibited RSD values below 30% (**Supplementary Figures 2B. D**), supporting the reliability of the metabolomic dataset. To evaluate overall metabolic differences among groups, multivariate analyses were performed using PCA and PLS-DA. In negative ion mode, PCA showed partial separation among the EMT, Benign, and Healthy groups (**Figure 4A**), which became more distinct under supervised PLS-DA modeling (**Figure 4B**). Permutation testing supported model robustness without evidence of overfitting (R² = 0.96, Q² = 0.52; **Figure 4C**). Similar patterns were observed in positive ion mode, where PCA showed group-level separation (**Figure 4D**), and PLS-DA further improved discrimination (R² = 0.99, Q² = 0.68; **Figures 4E, F**).

**Figure 5:**
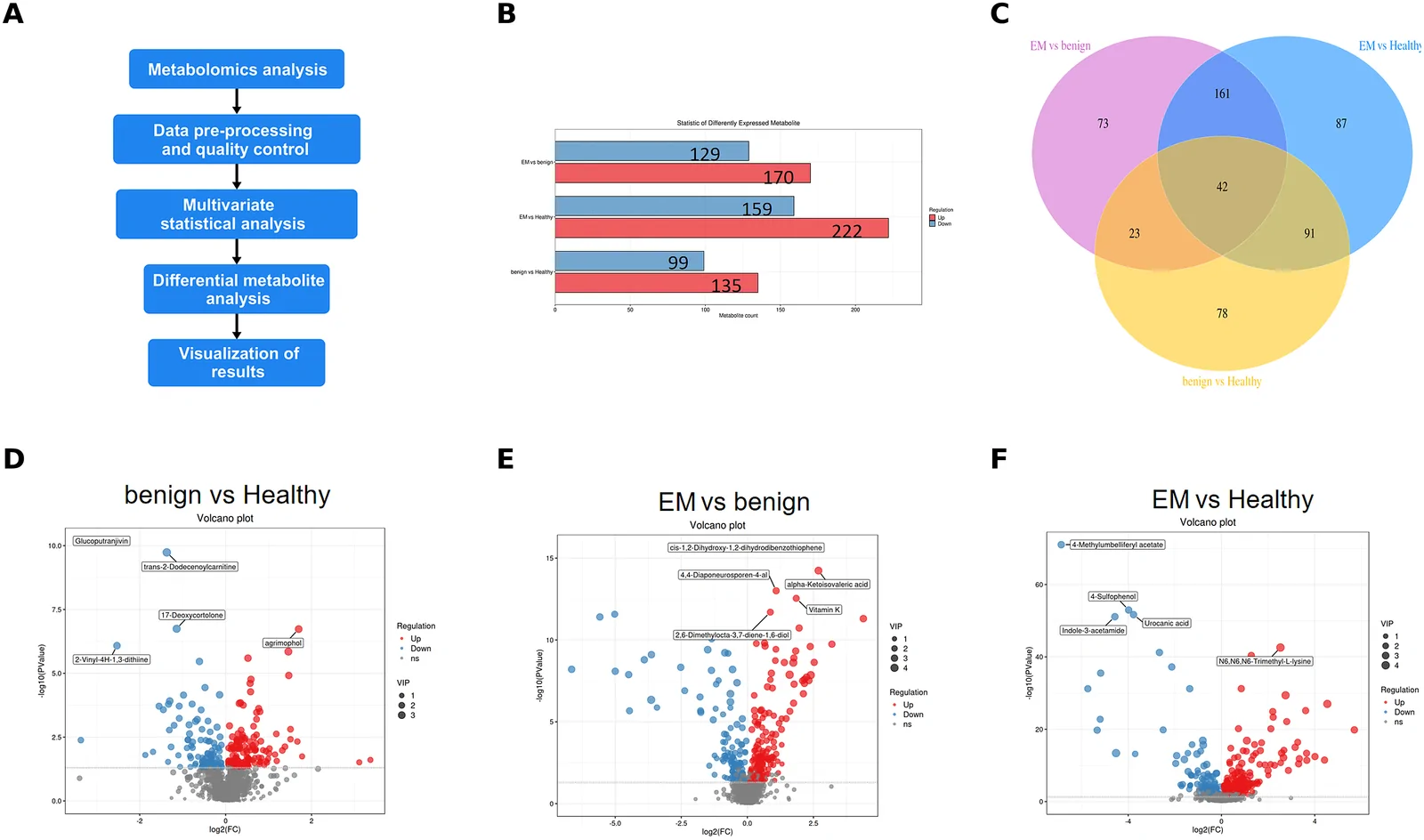
Comparative metabolomic profiling reveals differential metabolic signatures among healthy, benign, and EM groups. **(A)** Schematic overview of the metabolomics analysis pipeline. **(B)** Distribution of significantly upregulated and downregulated metabolites identified in each pairwise comparison. **(C)** Venn diagram illustrating shared and unique differential metabolites among EM vs Benign, EM vs Healthy, and Benign vs Healthy comparisons. **(D–F)** Volcano plots showing significantly altered metabolites in Benign vs Healthy, EM vs Benign, and EM vs Healthy groups, respectively. Differential metabolites were identified based on fold-change and statistical significance criteria. Red and blue dots indicate upregulated and downregulated metabolites, respectively, whereas gray dots represent non-significant metabolites. Point size reflects the VIP score derived from multivariate analysis.

Differential metabolite analysis identified substantial metabolic alterations across all comparisons (**Figures 5A, B**). In the EM versus Benign comparison, 299 differential metabolites were identified, including 170 upregulated and 129 downregulated metabolites. In the EM versus Healthy comparison, 381 differential metabolites were detected, comprising 222 upregulated and 159 downregulated metabolites. In the Benign versus Healthy comparison, 234 differential metabolites were identified, including 135 upregulated and 99 downregulated metabolites. Venn diagram analysis demonstrated both shared and group-specific metabolic alterations across comparisons (**Figure 5C**). A total of 42 differential metabolites were common to all three comparisons, whereas the EM group showed the largest number of unique differential metabolites, indicating broader metabolic perturbation. Volcano plot analysis further illustrated the distribution and magnitude of differential metabolites across comparisons (**Figures 5D–F**). Distinct metabolite signatures were observed between all pairwise groups, supporting substantial metabolic remodeling associated with EMT. To assess annotation reliability, all 1,372 MS/MS-annotated metabolites were classified according to the Metabolomics Standards Initiative (MSI) framework (**Supplementary Figure 3**). Among these, 337 metabolites (24.6%) were assigned as MSI level 1, 712 (51.9%) as MSI level 2, and 323 (23.5%) as MSI level 3, indicating that most candidate metabolites were supported by moderate-to-high annotation confidence. These results demonstrate widespread systemic metabolic alterations in EMand provide a molecular basis for subsequent pathway enrichment and integrative multi-omics analyses.

### 3.5 Candidate metabolite evaluation reveals discriminatory signatures in endometriosis and benign ovarian lesions

**Figure 6:**
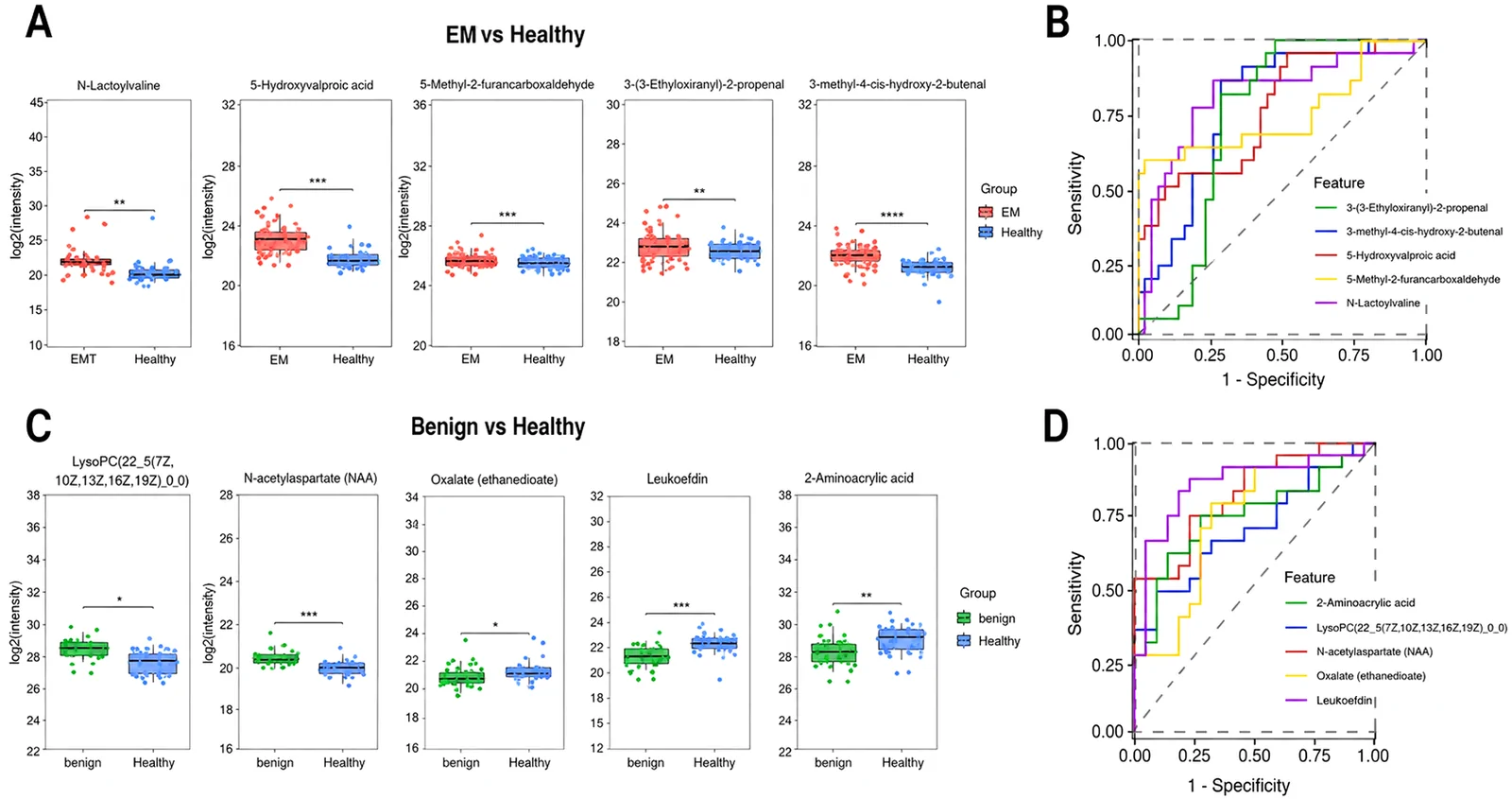
Differential metabolite signatures and their diagnostic utility. **(A, C)** Representative metabolites exhibiting significant abundance differences between EMand Healthy groups **(A)** and between Benign and Healthy groups **(C)**. **(B, D)** ROC curve analyses assessing the discriminatory power of the selected metabolites for EM versus Healthy **(B)** and Benign versus Healthy **(D)** classification. Together, these results identify candidate metabolite biomarkers with potential diagnostic value. * p < 0.05; ** p < 0.01; *** p < 0.001 **** p < 0.0001.

To further assess the discriminatory potential of selected differential metabolites, representative candidate metabolites were prioritized for group-wise evaluation based on differential abundance and receiver operating characteristic (ROC) analysis. In the EM versus Healthy comparison, five candidate metabolites showed significant abundance differences (**Figure 6A**). N-Lactoylvaline, 5-Hydroxyvalproic acid, and 3-methyl-4-cis-hydroxy-2-butenal were significantly elevated in the EM group, whereas 3-(3-Ethyloxiranyl)-2-propenal and 5-Methyl-2-furancarboxaldehyde were significantly decreased. ROC analysis demonstrated that all five metabolites showed moderate-to-good discriminatory performance (**Figure 6B**), with N-Lactoylvaline showing the highest AUC among the evaluated metabolites. In the Benign versus Healthy comparison, five representative metabolites also showed significant abundance differences (**Figure 6C**). LysoPC(22:5), N-acetylaspartate (NAA), and oxalate (ethanedioate) showed increased abundance in the Benign group, whereas Leukoefdin and 2-aminoacrylic acid showed reduced abundance. ROC analysis demonstrated favorable discriminatory performance for these metabolites (**Figure 6D**), with Leukoefdin showing the highest AUC in this comparison. Using the Olink Target 96 Inflammation panel, we performed serum proteomic profiling in 88 participants, including Benign (n = 22), EM (n = 44), and Healthy (n = 22). To improve statistical robustness, ROC analyses were further evaluated using 2,000 stratified bootstrap iterations with feature-wise Benjamini-Hochberg FDR correction across all 1,464 molecular features (**Supplementary Figures 4**, **S5**). In the EM versus Healthy comparison (**Supplementary Figure 4**), all five prespecified metabolites retained significant discriminatory performance after global correction, with AUC values ranging from 0.748 to 0.815 (**Table 1**). Among these, N-Lactoylvaline showed the strongest performance (AUC = 0.815, 95% CI 0.698–0.925, FDR = 3.38 × 10^-4^). In the Benign versus Healthy comparison (**Supplementary Figure 5**), Leukoefdin (AUC = 0.849, 95% CI 0.717–0.961, FDR = 0.008) and N-acetylaspartate (AUC = 0.812, 95% CI 0.669–0.917, FDR = 0.022) remained significant after correction, whereas the remaining metabolites showed nominal but non-significant performance after multiple testing adjustment. These results identify distinct metabolite signatures associated with EMand benign ovarian lesions and support their prioritization for subsequent integrative multi-omics modeling.

**Table 1:** Diagnostic performance of differential metabolites identified in EM and benign groups compared with healthy controls.

Feature	AUC
N-Lactoylvaline	0.815082645
3-methyl-4-cis-hydroxy-2-butenal	0.789256198
5-Hydroxyvalproic acid	0.771694215
5-Methyl-2-furancarboxaldehyde	0.756198347
3-(3-Ethyloxiranyl)-2-propenal	0.747933884
2-Aminoacrylic acid	0.729338843
LysoPC(22_5(7Z,10Z,13Z,16Z,19Z) _0_0)	0.688016529
N-acetylaspartate (NAA)	0.811983471
Oxalate (ethanedioate)	0.719008264
Leukoefdin	0.849173554

### 3.6 Metabolic pathway enrichment reveals activation of the retinoic acid-IgA axis and amino acid-carbon metabolic reprogramming in EMT

**Figure 7:**
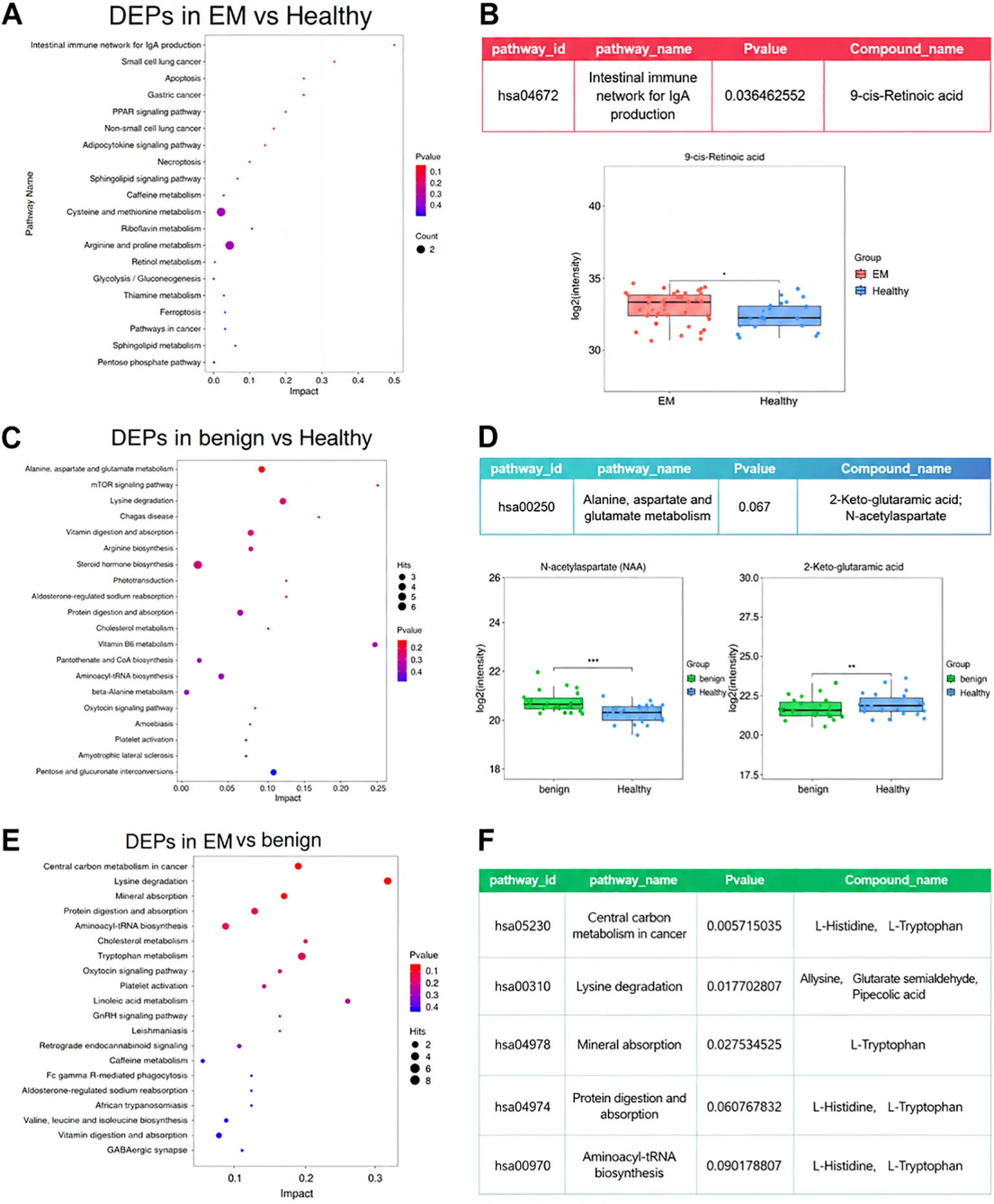
Pathway enrichment analysis of EMT-associated differential metabolites reveals immune-metabolic coupling mechanisms. **(A)** KEGG pathway enrichment bubble plot of differential metabolites in the EM vs. Healthy group; **(B)** Analysis of the “Intestinal IgA Immune Network” pathway and its key metabolite, 9-cis-Retinoic acid; **(C)** KEGG pathway enrichment bubble plot of differential metabolites between the Benign and Healthy groups; **(D)** Expression distribution of metabolites involved in the alanine-aspartate-glutamate metabolism pathway; **(E)** KEGG pathway enrichment bubble plot of differential metabolites in the EM vs. Benign comparison; **(F)** Tabulated summary of significantly enriched pathways and corresponding differential metabolites between EMand Benign groups. * p < 0.05; ** p < 0.01; *** p < 0.001.

To further elucidate the metabolic regulatory mechanisms associated with EMT, we performed KEGG pathway enrichment analysis based on the significantly altered metabolites. The results indicated that differential metabolites in the EM vs. Healthy comparison were primarily enriched in several metabolic pathways, including retinol metabolism, riboflavin metabolism, arginine and proline metabolism, cysteine and methionine metabolism, and sphingolipid signaling (**Figure 7A**). The intestinal IgA immune network pathway (hsa04672) showed nominal enrichment (nominal *p* = 0.036), although it did not remain statistically significant after multiple-testing correction. Given its biological relevance and the presence of differential metabolites contributing to this pathway, including 9-cis-retinoic acid, it was considered a candidate immune-metabolic pathway requiring further validation (**Figure 7B**). Its expression was significantly elevated in EM patients (*p* < 0.05), indicating enhanced retinoic acid metabolic activity. Given the pivotal role of retinoic acid signaling in immune tolerance, epithelial differentiation, and local inflammation control, its upregulation may contribute to the immune-metabolic interplay in EM via the retinoic acid-IgA axis, thereby facilitating lesion establishment and the persistence of chronic inflammation. We conducted KEGG metabolic pathway enrichment analysis between the Benign and Healthy groups. The results showed that the differential metabolites were mainly enriched in pathways including alanine, aspartate, and glutamate metabolism; the mTOR signaling pathway; arginine biosynthesis; and steroid hormone biosynthesis (**Figure 7C**). Although the alanine-aspartate-glutamate metabolism pathway showed nominal enrichment (nominal p = 0.067) and did not meet the predefined FDR significance threshold, it involved multiple metabolites with significant abundance changes, including NAA and α-ketoglutarate, suggesting a potential metabolic alteration that warrants further investigation (**Figure 7D**), suggesting that this pathway may represent a shared dysregulated metabolic axis during the progression from benign lesions to EM. Further quantitative analysis revealed that NAA was significantly upregulated in the Benign group, whereas α-ketoglutarate was markedly downregulated (**Figure 7D**). The increase in NAA may reflect enhanced neurogenic amino acid metabolism and accumulation of intermediates within the tricarboxylic acid (TCA) cycle, while the reduction in α-ketoglutarate indicates a constrained metabolic flux within energy metabolism.

To clarify the specific metabolic alterations in the EM group compared to the Benign group, KEGG pathway enrichment analysis was performed on the differential metabolites between the two groups. As shown in **Figure 7E**, the significantly enriched pathways included central carbon metabolism in cancer, lysine degradation, mineral absorption, protein digestion and absorption, and aminoacyl-tRNA biosynthesis. Among these, central carbon metabolism in cancer exhibited the most significant enrichment (*p* = 0.0057), involving key metabolites such as L-histidine and L-tryptophan (**Figure 7F**). The upregulation of these amino acids suggests a reallocation of carbon flow and amino acid metabolic flux, indicating that EM lesions exhibit tumor-like features characterized by elevated anabolic activity and increased energy demands. In addition, the enrichment of the lysine degradation and aminoacyl-tRNA biosynthesis pathways further indicates activation of protein metabolism and amino acid cycling within EM tissues. Our results suggest that EM is associated with a distinct pattern of metabolic reprogramming, characterized by activation of the retinoic acid-IgA signaling axis, enhanced amino acid and carbon metabolic flux, and suppressed mitochondrial energy metabolism.

### 3.7 Integrative multi-omics analysis reveals coordinated immune–metabolic associations in endometriosis

**Figure 8:**
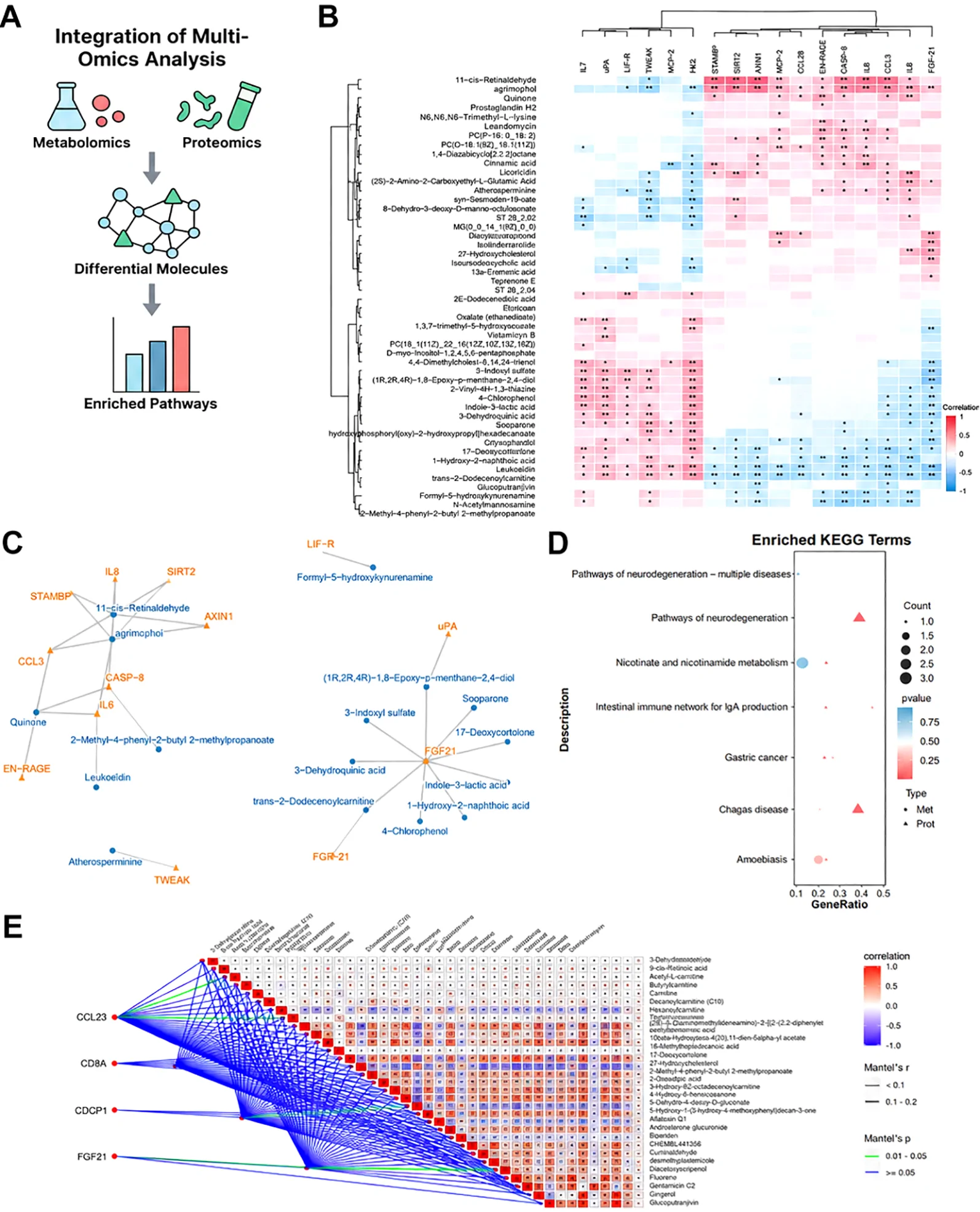
Multi-omics correlation analysis reveals systemic interactions between inflammatory signals and metabolic reprogramming. **(A)** Workflow of proteome-metabolome integrative analysis; **(B)** Pearson correlation heatmap of the top 50 differential molecules, illustrating molecular-level associations between inflammation-related proteins and metabolites; **(C)** Protein-metabolite interaction network constructed from significantly correlated molecule pairs (|r| > 0.4, p < 0.05), highlighting interconnections across major functional modules; **(D)** Joint KEGG pathway enrichment analysis of proteomic and metabolomic data, showing enrichment of differential molecules in shared biological pathways; **(E)** Mantel multi-omics correlation analysis between the proteome and metabolome. * p < 0.05; ** p < 0.01.

To investigate the coordinated relationships between differential proteins and metabolites, integrative multi-omics analyses were performed using correlation-based network modeling, joint pathway enrichment, and Mantel association analysis (**Figure 8A**). Pearson correlation analysis of the top-ranked differential proteins and metabolites identified extensive protein-metabolite associations (**Figure 8B**). Several inflammatory proteins, including IL8, IL6, CCL3, CASP-8, and EN-RAGE, showed significant correlations with metabolites involved in lipid metabolism, redox balance, and energy-associated pathways (|r| > 0.4, *p* < 0.05). Strong positive correlations were observed between agrimophol, 11-cis-retinaldehyde, and quinone with proteins including AXIN1, SIRT2, and STAMBP, whereas several metabolites, including L-glutamic acid and oxalate, showed inverse correlations with these proteins. Protein-metabolite interaction network analysis demonstrated modular organization of correlated molecular features (**Figure 8C**). The network revealed multiple interconnected hubs linking inflammatory proteins and metabolite clusters, supporting structured immune-metabolic connectivity within the circulating molecular landscape. Joint KEGG enrichment analysis across proteomic and metabolomic datasets identified several shared pathways (**Figure 8D**), including nicotinate and nicotinamide metabolism, intestinal immune network for IgA production, and broader signaling pathways represented across both omics layers. These shared enrichments indicate overlapping functional domains between inflammatory signaling and metabolic regulation. To further assess system-level associations, Mantel correlation analysis was performed using differential proteins and metabolites (**Figure 8E**). The integrative network identified molecular clusters centered around FGF21, CDCP1, CD8A, and CCL23, suggesting that these proteins represent candidate nodes associated with coordinated immune-metabolic patterns. However, the Mantel correlation coefficients reflected modest global associations between molecular layers rather than strong direct molecular correlations (**Supplementary Table 1**). Within the FGF21-centered module, fluorene showed a positive association (r = 0.142, *p* = 0.012), while in the CCL23-centered module, acetyl-L-carnitine showed a positive correlation (r = 0.15, *p* = 0.019). Additional weaker correlations with tiglylcarnitine and related acylcarnitines were also observed. In contrast, CD8A and CDCP1 showed comparatively fewer significant metabolite associations.

To determine whether these associations were independent of diagnostic grouping, group-adjusted correlation analysis was performed by residualizing both proteomic and metabolomic data for group effects (**Supplementary Figure 6**). A total of 268 protein-metabolite pairs remained significant after FDR correction. The strongest adjusted associations included AXIN1–agrimophol (r = 0.767, FDR = 3.71 × 10^-^13), AXIN1–11-cis-retinaldehyde (r = 0.757, FDR = 8.58 × 10^-^13), SIRT2–agrimophol (r = 0.727, FDR = 4.57 × 10^-^11), and IL8–agrimophol (r = 0.671, FDR = 1.82 × 10^-^8), indicating that part of the observed immune-metabolic coupling persisted beyond group-level separation. These analyses demonstrate coordinated associations between inflammatory proteins and circulating metabolites in EM, supporting the presence of systemic immune-metabolic coupling and prioritizing FGF21, CCL23, CDCP1, and CD8A as central nodes for downstream diagnostic and mechanistic evaluation.

### 3.8 Multi-omics integration improves diagnostic classification performance in EMT

**Figure 9:**
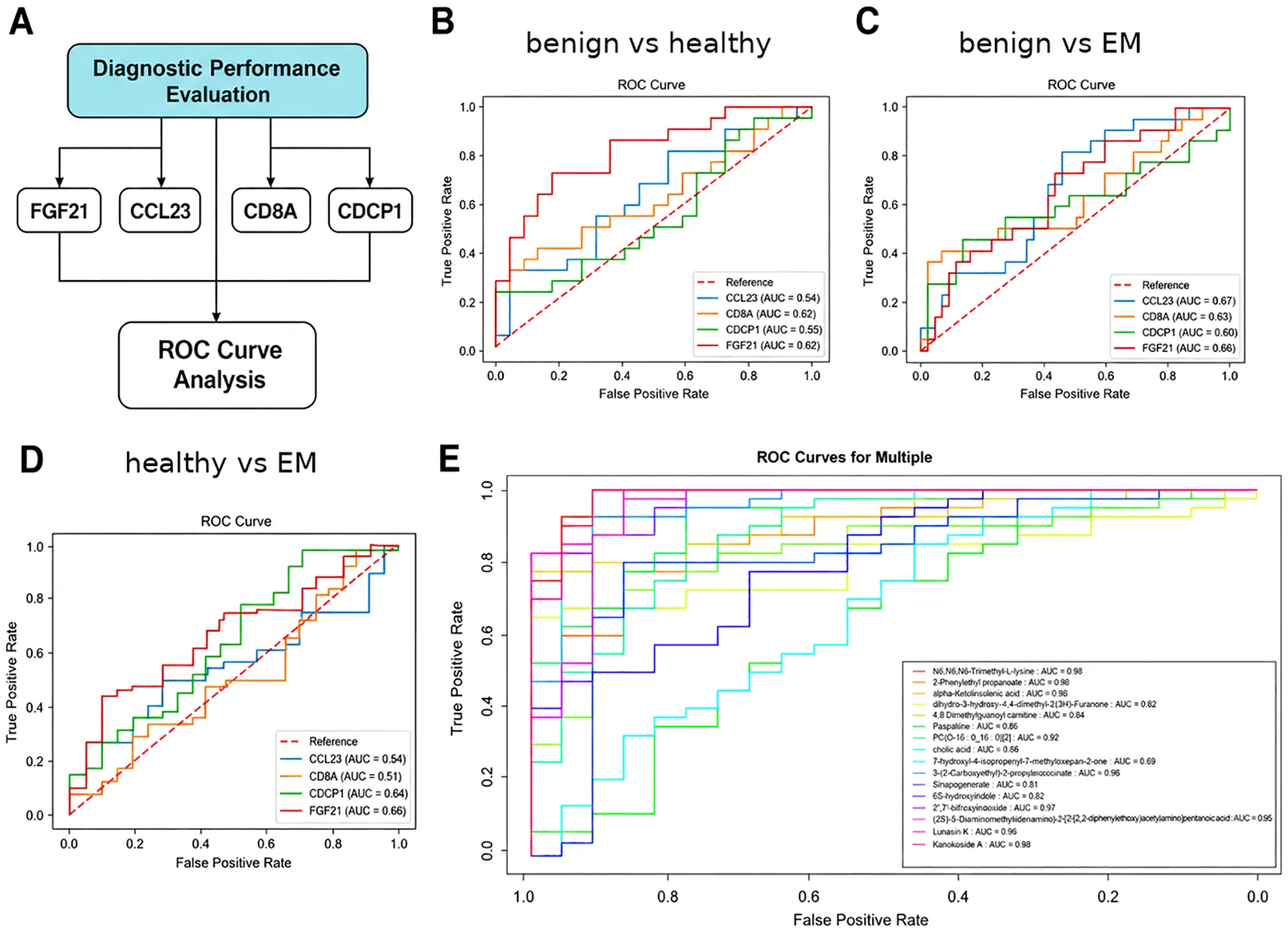
Diagnostic evaluation of candidate protein and metabolite biomarkers. **(A)** Workflow of ROC-based diagnostic performance analysis using selected protein biomarkers. **(B–D)** ROC curves for CCL23, CD8A, CDCP1, and FGF21 in Benign versus Healthy, Benign versus EMT, and Healthy versus EM comparisons, respectively. **(E)** ROC analysis of candidate metabolite biomarkers identified through multi-omics profiling, demonstrating their ability to discriminate among clinical groups and highlighting metabolites with high diagnostic accuracy.

To evaluate the diagnostic performance of key proteins identified through integrative multi-omics analysis, ROC analyses were performed for FGF21, CCL23, CD8A, and CDCP1 across the three pairwise comparisons (**Figure 9A**). In the benign versus Healthy comparison (**Figure 9B**), FGF21 showed the highest discriminatory performance among the four proteins in the original ROC analysis (AUC = 0.62). Bootstrap resampling demonstrated variability in model performance, with a mean bootstrap-derived AUC of 0.816 (FDR = 0.019), suggesting that the estimated performance was sensitive to sampling variation and should be interpreted as internally validated performance rather than an improvement of the observed AUC. These findings indicate that FGF21 contributed the strongest single-protein signal in distinguishing benign ovarian lesions from Healthy controls. In the benign versus EM comparison (**Figure 9C**), CCL23 demonstrated the highest classification performance (AUC = 0.67), followed by FGF21 (AUC = 0.66), CD8A (AUC = 0.63), and CDCP1 (AUC = 0.60). Although these effect sizes were moderate, they suggest that inflammatory and metabolic proteins together capture part of the molecular distinction between benign and EM samples. The Random Forest model integrating multiple molecular features achieved high internal classification performance between benign and Healthy groups (AUC = 0.960). This improved performance compared with individual proteins suggests that the combined model captures complementary molecular information. However, given the exploratory design and absence of external validation, this performance estimate requires further confirmation in independent cohorts. In the Healthy versus EM comparison (**Figure 9D**), FGF21 again showed the strongest individual discrimination (AUC = 0.66), followed by CDCP1 (AUC = 0.64), whereas CCL23 (AUC = 0.54) and CD8A (AUC = 0.51) showed limited standalone performance. Bootstrap-adjusted analyses confirmed that FGF21 retained modest but stable classification ability, whereas the remaining proteins showed weaker corrected performance.

To assess whether multi-marker integration improved classification accuracy, combined ROC models incorporating differential metabolites and proteins were evaluated (**Figure 9E**). Compared with single-protein models, multi-marker panels demonstrated substantially improved discrimination, with several metabolite-rich models achieving AUC values above 0.90. Among these, N6, N6, N6-trimethyl-L-lysine, Kanokoside A, and α-hydroxyisocaproate showed the highest individual performance, with AUCs ranging from 0.96 to 0.98. Internal validation using repeated stratified five-fold cross-validation further supported the robustness of these models (**Supplementary Figure 7**). For EMversus Healthy, the five-metabolite panel achieved a mean cross-validated AUC of 0.829 ± 0.021, whereas the combined protein-metabolite panel yielded a comparable AUC of 0.817 ± 0.038. In the benign versus Healthy comparison, the combined panel showed the strongest performance, with a mean cross-validated AUC of 0.960 ± 0.022. In contrast, the four-protein panel alone showed weaker classification performance for EMversus Healthy (AUC = 0.571 ± 0.070) and moderate performance for EMversus benign (AUC = 0.678 ± 0.027). These results indicate that metabolite-based and integrated multi-omics models outperform single protein biomarkers for classifying EMand benign ovarian lesions in the present cohort. While proteins such as FGF21 and CCL23 contribute informative signals, their individual diagnostic performance remains limited, supporting their use as part of broader multi-marker models rather than standalone clinical biomarkers. To further evaluate the clinical relevance of the identified molecular signatures, we assessed their relationship with serum CA125 levels, a commonly used biomarker in endometriosis. Spearman correlation analysis demonstrated that serum CA125 levels were not significantly correlated with the core protein markers (FGF21, CCL23, CD8A, and CDCP1) or the candidate metabolite signatures after FDR correction (adjusted *p >*0.05). These findings suggest that the identified immunometabolic alterations provide complementary molecular information beyond conventional CA125 assessment.

## 4 Discussion

This study systematically characterized the inflammatory and metabolic networks in the peripheral blood of patients with EMby integrating Olink-based inflammatory proteomics with untargeted metabolomics data. It identified a core metabolic-immune interaction module composed of FGF21, CCL23, CD8A, and CDCP1, and demonstrated its potential diagnostic and subtyping value in EMT. The findings revealed that the pathogenesis of EM involves not only localized inflammation and immune dysregulation, but also systemic metabolic reprogramming. These results suggest that EM represents a systemic immune-metabolic disorder rather than a disease confined to ectopic lesions.

In the present study, we performed an integrated inflammatory proteomic and untargeted metabolomic analysis of peripheral blood from patients with EM, benign ovarian cysts, and healthy controls to characterize systemic molecular alterations associated with EM. By combining Olink inflammatory proteomics, untargeted LC-MS metabolomics, pathway enrichment, machine learning-based feature prioritization, and multi-omics correlation modeling, we identified a coordinated immune-metabolic signature centered on FGF21, CCL23, CD8A, and CDCP1. These findings support the concept that EMis associated not only with local ectopic inflammatory activity but also with systemic immune-metabolic remodeling (20, 21). Importantly, the integration approach used in this study was not limited to a single strategy. Instead, we applied a layered framework in which pathway-based analyses provided biological context, correlation-based networks captured molecular connectivity, and predictive models evaluated the discriminatory contribution of individual molecular features. This complementary strategy allowed both mechanistic interpretation and biomarker prioritization within the same analytical framework. Proteomic profiling revealed a clear inflammatory phenotype in EM. Differential expression analysis showed significant upregulation of multiple inflammatory mediators, particularly chemokines and apoptosis-related proteins, including CCL23, CASP-8, EN-RAGE, CCL3, and FGF23, while FGF21 was consistently downregulated. Importantly, overlap analysis demonstrated that many inflammatory alterations identified in benign ovarian cysts were also present in EM, but EM exhibited additional disease-specific signals, suggesting progressive amplification of inflammatory signaling during disease evolution. This pattern supports the notion that EM shares common inflammatory features with benign gynaecological lesions but develops into a more complex immune-regulatory state (20, 22). Functional enrichment analysis further reinforced this inflammatory framework. GO analysis showed significant enrichment in granulocyte activation, eosinophil migration, leukocyte chemotaxis, and natural killer cell recruitment, indicating that immune-cell trafficking represents a dominant systemic feature of EMT. KEGG and Reactome analyses consistently converged on chemokine signaling, cytokine-receptor interaction, interleukin signaling, and caspase-mediated apoptosis pathways. Notably, the enrichment of CCR-related pathways corresponded with elevated CCL23 expression. Since CCL23 is a known ligand of CCR1 and mediates monocyte and macrophage recruitment (23, 24), this finding suggests that CCR1-dependent immune-cell trafficking may contribute to persistent inflammatory activation in EM lesions.

Machine learning-based feature prioritization identified CD8A, CCL23, and FGF21 as the most discriminative proteins between EMand benign lesions. These molecules collectively represent three biologically interconnected axes: immune activation (CD8A), inflammatory chemotaxis (CCL23), and metabolic adaptation (FGF21). CD8A elevation may reflect altered systemic cytotoxic T-cell recruitment; however, its relatively modest diagnostic performance and limited metabolite associations suggest that peripheral CD8+ T-cell activity may only partially reflect the local immune microenvironment. This observation is consistent with previous studies showing functional exhaustion and impaired cytotoxicity of CD8+ T cells in endometriosis lesions (24, 25). Untargeted metabolomics revealed extensive metabolic reprogramming in EM. Compared with healthy controls and benign ovarian lesions, EM exhibited the highest number of differential metabolites, suggesting broad metabolic perturbation. PCA and PLS-DA analyses demonstrated clear metabolic separation among groups, supporting distinct disease-associated metabolic phenotypes. Several EMT-associated metabolites including N-lactoylvaline, 5-hydroxyvalproic acid, and 3-methyl-4-cis-hydroxy-2-butenal showed significant differential abundance and favorable diagnostic performance. These metabolites are associated with glycolytic flux, short-chain fatty acid metabolism, and oxidative stress adaptation, suggesting that EM is accompanied by altered energy utilization and redox imbalance (26, 27). Among the identified proteins, FGF21 emerged as a central metabolic regulator. FGF21 is a well-established endocrine factor involved in glucose homeostasis, fatty acid β-oxidation, ketogenesis, and mitochondrial adaptation to metabolic stress (28, 29). Interestingly, unlike many metabolic-inflammatory disorders where FGF21 is elevated as a compensatory response, we observed significantly reduced circulating FGF21 in EMT. This may indicate impaired metabolic compensation or insufficient mitochondrial stress adaptation. FGF21 also exhibited strong positive correlations with metabolites involved in retinoid metabolism and redox regulation, including 11-cis-retinaldehyde, agrimophol, and quinone derivatives. Retinoid metabolism has been implicated in oxidative stress adaptation and immune regulation (30), suggesting that FGF21 may function as a central buffering node linking metabolic stress and inflammatory signaling.

CCL23 represented the strongest inflammatory-metabolic connector identified in this study. CCL23 is a chemokine that mediates monocyte and macrophage chemotaxis through CCR1 signaling and is associated with chronic inflammatory diseases (23). In our data, CCL23 showed significant positive correlations with acetyl-L-carnitine and tiglylcarnitine, both key intermediates in mitochondrial fatty acid transport and β-oxidation (31). Acylcarnitines are essential for shuttling long-chain fatty acids into mitochondria for oxidation, and their association with CCL23 suggests a coordinated relationship between inflammatory activation and mitochondrial energy metabolism. This finding provides a potential mechanistic link between chronic chemokine signaling and altered lipid utilization in EM. Multi-omics integration further revealed coordinated protein-metabolite coupling beyond diagnostic group separation. Several strong correlations persisted after adjusting for group effects, particularly involving AXIN1, SIRT2, IL8, and STAMBP with retinaldehyde- and agrimophol-related metabolites. AXIN1 is a key regulator of Wnt/β-catenin signaling, which has been implicated in endometriosis lesion growth and fibrosis (32). SIRT2 is involved in mitochondrial stress responses and redox homeostasis. These findings suggest convergence between inflammatory signaling, oxidative metabolism, and tissue remodeling pathways, supporting the concept that EM-associated systemic alterations are highly interconnected.

From a diagnostic perspective, our data demonstrate that metabolite-based signatures outperform single inflammatory proteins. While the four-protein panel (FGF21, CCL23, CD8A, CDCP1) showed only moderate discriminatory ability, metabolite panels consistently achieved higher AUC values, and integrated multi-omics models showed the strongest classification performance. This suggests that metabolic alterations may represent more stable and robust peripheral signatures than inflammatory proteins alone. Previous metabolomics studies have similarly reported strong biomarker potential for circulating metabolic profiles in endometriosis (33). Biologically, these findings support a model in which EM progression is sustained by reciprocal interactions between inflammatory chemokine recruitment and metabolic adaptation. Elevated CCL23 promotes immune-cell trafficking, apoptosis-related signaling contributes to lesion remodeling, and reduced FGF21 may impair metabolic buffering capacity. Together, these processes may promote persistent oxidative stress, altered fatty acid oxidation, and mitochondrial dysfunction, thereby facilitating lesion survival and chronic disease progression. The observed alterations in FGF21, acylcarnitine metabolism, and inflammatory chemokines may reflect broader immunometabolic processes also described in obesity-associated inflammation, metabolic syndrome, and other chronic inflammatory conditions, supporting the concept of endometriosis as part of a systemic inflammatory continuum. Although FGF21, CCL23, CD8A, and CDCP1 emerged as prioritized network-associated features, their individual diagnostic performance was modest, indicating that these proteins should not be interpreted as standalone biomarkers. The observed Mantel associations represent coordinated patterns between proteomic and metabolomic landscapes rather than direct molecular correlations. Therefore, these molecules are considered candidate regulators or markers of immune-metabolic remodeling that require further functional and external validation. The apparent difference between original and bootstrap-derived AUC estimates reflects internal resampling variability rather than an enhancement of biomarker performance. Therefore, FGF21 should be considered a candidate molecular feature requiring validation in independent cohorts rather than a standalone diagnostic marker.

This study provides a comprehensive cross-sectional multi-omics framework for characterizing systemic immune-metabolic alterations associated with EMand establishes a foundation for future mechanistic and translational investigations. The integrated proteomic and metabolomic signatures identified here represent candidate molecular networks that warrant further validation using orthogonal quantitative approaches, including ELISA and targeted LC-MS/MS, to strengthen analytical precision and clinical applicability. Although disease stage information was available based on rASRM classification, stage-stratified multi-omics analyses were not performed because of the limited sample size within individual severity categories. Since inflammatory and metabolic alterations may vary across disease stages, future studies involving larger, clinically stratified cohorts are required to determine whether the identified immunometabolic signatures are associated with disease progression or severity. Future studies incorporating larger, clinically stratified cohorts and external validation datasets will be important for refining these candidate biomarkers and evaluating their performance across disease subtypes and progression stages. Building on these findings, future research should prioritize functional investigation of the FGF21–CCL23 immunometabolic axis, targeted quantification of acylcarnitine and redox-associated metabolites, and longitudinal monitoring to assess their relationship with disease progression, recurrence, and therapeutic response. Furthermore, integration with emerging technologies such as single-cell multi-omics and spatial transcriptomics will provide higher-resolution insights into the cellular origin, spatial organization, and functional dynamics of these circulating immune-metabolic signatures, thereby advancing both mechanistic understanding and precision diagnostic development in EM. Another limitation is that serum rather than plasma was used for metabolomic profiling. Although serum is commonly applied for biomarker discovery studies and provides a robust representation of circulating molecular changes, the coagulation process during serum preparation may influence the abundance of certain platelet-derived inflammatory mediators and metabolites. Therefore, some detected molecular alterations should be interpreted as serum-associated signatures rather than direct measurements of circulating plasma concentrations. Future studies using matched serum and plasma samples will be valuable for validating the biological specificity of the identified immunometabolic signatures. In addition, although menstrual cycle phase was recorded and recent hormonal therapy was excluded, residual effects of physiological hormonal fluctuations cannot be completely eliminated. Since estrogen and progesterone dynamics may influence immune activity and metabolic pathways, future studies with larger cohorts and standardized sampling during defined menstrual cycle phases will be important to further validate the identified immunometabolic signatures. In addition to sample size limitations, potential biological and clinical confounding factors should be considered when interpreting the identified immunometabolic signatures. Although participants were selected using predefined inclusion and exclusion criteria and recent hormonal therapy was excluded, residual effects of age, body composition, menstrual cycle-related hormonal fluctuations, medication exposure, and other metabolic factors may influence circulating inflammatory and metabolomic profiles. Furthermore, endometriosis represents a biologically heterogeneous disorder comprising distinct phenotypes, including superficial peritoneal lesions, ovarian endometriomas, and deep infiltrating disease, which may exhibit different immune-metabolic characteristics. Because our cohort size limited detailed subgroup analyses according to lesion subtype and disease severity, future studies with larger multicenter cohorts and comprehensive clinical stratification will be required to determine the generalizability and subtype specificity of the identified signatures. Because serum preparation may introduce platelet-derived contributions compared with plasma, future studies using matched serum and plasma samples will be important to determine whether the identified signatures are matrix-dependent.

## 5 Conclusion

The integrated inflammatory proteomic and metabolomic profiling of peripheral blood revealed coordinated immune-metabolic alterations associated with endometriosis. Multi-omics analyses identified a candidate immunometabolic signature involving FGF21, CCL23, CD8A, and CDCP1, linking inflammatory signaling with metabolic pathways involved in fatty acid utilization, redox regulation, and cellular stress responses. Proteomic analyses highlighted dysregulation of chemokine- and cytokine-related pathways, whereas metabolomic profiling demonstrated widespread metabolic remodeling, supporting the presence of systemic immune-metabolic perturbations in endometriosis. Integrative network and correlation analyses further revealed coordinated protein-metabolite associations, suggesting functional interplay between inflammatory activation and metabolic adaptation. From a translational perspective, metabolite-rich and combined multi-omics models showed improved classification performance compared with individual protein markers, supporting the potential value of blood-based immunometabolic signatures for future biomarker development. Collectively, these findings expand current understanding of the systemic molecular landscape of endometriosis and provide a framework for future mechanistic studies, biomarker validation, and precision diagnostic approaches. Further investigation in larger, independent cohorts and targeted experimental studies will be important to determine the biological and clinical relevance of these candidate immune-metabolic signatures.

## Data Availability

The datasets presented in this study can be found in online repositories. The names of the repository/repositories and accession number(s) can be found in the article/**Supplementary Material**.

## References

[1] Blamble T Dickerson L . Recognizing and treating endometriosis. Jaapa. (2021) 34:14–9. doi: 10.1097/01.JAA.0000750940.47126.58 34031308

[2] Czubak P Herda K Niewiadomska I Putowski L Łańcut M Masłyk M . Understanding endometriosis: A broad review of its causes, management, and impact. Int J Mol Sci. (2025) 26:8878. doi: 10.3390/ijms26188878 41009445 PMC12469443

[3] Abike F Tanoglu FB Sidar G . Deep pelvic endometriosis: clinical features, diagnosis, and treatment - a comprehensive review. Arch Gynecol Obstet. (2025) 312:1857–69. doi: 10.1007/s00404-025-08187-0 41026192 PMC12705831

[4] Li Y-F Li W-W Yang X-H Yang Y-B Ye Q-J . Clinical characteristics of endometriosis with and without dysmenorrhea diagnosed by laparoscopy. Front Med (Lausanne). (2025) 12:1635960. doi: 10.3389/fmed.2025.1635960 41020245 PMC12462631

[5] Moradi Y Shams-Beyranvand M Khateri S Gharahjeh S Tehrani S Varse F . A systematic review on the prevalence of endometriosis in women. Indian J Med Res. (2021) 154:446–54. doi: 10.4103/ijmr.IJMR_817_18 35345070 PMC9131783

[6] Nisenblat V Bossuyt PMM Shaikh R Farquhar C Jordan V Scheffers CS . Blood biomarkers for the non-invasive diagnosis of endometriosis. Cochrane Database Syst Rev. (2016) 2016:CD012179. doi: 10.1002/14651858.CD012179 27132058 PMC7076288

[7] Shokrnejad-Namin T Farzizadeh N Najmi Z Amoozgar M Hariri A Amini E . Evaluating the diagnostic potential of CA-125 and miRNA levels in endometriosis: A narrative review. Int J Gynaecol Obstet. (2026) 172:1392–429. doi: 10.1002/ijgo.70554 41031526

[8] Ding Z Wang N Ji N Chen Z-S . Proteomics technologies for cancer liquid biopsies. Mol Cancer. (2022) 21:53. doi: 10.1186/s12943-022-01526-8 35168611 PMC8845389

[9] Bujak R Struck-Lewicka W Markuszewski MJ Kaliszan R . Metabolomics for laboratory diagnostics. J Pharm BioMed Anal. (2015) 113:108–20. doi: 10.1016/j.jpba.2014.12.017 25577715

[10] Schrimpe-Rutledge AC Codreanu SG Sherrod SD McLean JA . Untargeted metabolomics strategies-challenges and emerging directions. J Am Soc Mass Spectrom. (2016) 27:1897–905. doi: 10.1007/s13361-016-1469-y 27624161 PMC5110944

[11] Kharb S Joshi A . Multi-omics and machine learning for the prevention and management of female reproductive health. Front Endocrinol (Lausanne). (2023) 14:1081667. doi: 10.3389/fendo.2023.1081667 36909346 PMC9996332

[12] Chu T Cui J Sun L Zhang X Sun L Tong J . The disordered extracellular matrix landscape induced endometrial fibrosis of sheep: A multi-omics integrative analysis. Int J Biol Macromol. (2024) 265:130845. doi: 10.1016/j.ijbiomac.2024.130845 38503376

[13] Anchan MM Dutta R . Reframing endometriosis: Interplay of NETs, macrophages, and lymphocytes at the crossroads of disease progression, infertility, and Malignant transformation. Am J Reprod Immunol. (2025) 94:e70179. doi: 10.1111/aji.70179 41146532

[14] Kobayashi H Imanaka S . Understanding the molecular mechanisms of macrophage polarization and metabolic reprogramming in endometriosis: A narrative review. Reprod Med Biol. (2022) 21:e12488. doi: 10.1002/rmb2.12488 36310658 PMC9596393

[15] Scutiero G Iannone P Bernardi G Bonaccorsi G Spadaro S Volta CA . Oxidative stress and endometriosis: A systematic review of the literature. Oxid Med Cell Longev. (2017) 2017:7265238. doi: 10.1155/2017/7265238 29057034 PMC5625949

[16] Ni C Li D . Ferroptosis and oxidative stress in endometriosis: A systematic review of the literature. Med (Baltimore). (2024) 103:e37421. doi: 10.1097/MD.0000000000037421 38489713 PMC10939684

[17] Yang Q Guo C Zhang L . The role of metabolite sensors in metabolism-immune interaction: New targets for immune modulation. Clin Transl Med. (2025) 15:e70294. doi: 10.1002/ctm2.70294 40159576 PMC11955300

[18] Lang R Siddique MNAA . Control of immune cell signaling by the immuno-metabolite itaconate. Front Immunol. (2024) 15:1352165. doi: 10.3389/fimmu.2024.1352165 38487538 PMC10938597

[19] Tu X Tu M Xu J . Clinical application of peripheral blood biomarkers for solid tumors. Medcomm (2020). (2026) 7:e70654. doi: 10.1002/mco2.70654 41799938 PMC12960069

[20] Zondervan KT Becker CM Koga K Missmer SA Taylor RN Viganò P . Endometriosis. Nat Rev Dis Primers. (2018) 4:9. doi: 10.1038/s41572-018-0008-5 30026507

[21] García-Gómez E Vázquez-Martínez ER Reyes-Mayoral C Cruz-Orozco OP Camacho-Arroyo I Cerbón M . Regulation of inflammation pathways and inflammasome by sex steroid hormones in endometriosis. Front Endocrinol (Lausanne). (2019) 10:935. doi: 10.3389/fendo.2019.00935 32063886 PMC7000463

[22] Symons LK Miller JE Kay VR Marks RM Liblik K Koti M . The immunopathophysiology of endometriosis. Trends Mol Med. (2018) 24:748–62. doi: 10.1016/j.molmed.2018.07.004 30054239

[23] Patel VP Kreider BL Li Y Li H Leung K Salcedo T . Molecular and functional characterization of two novel human C-C chemokines as inhibitors of two distinct classes of myeloid progenitors. J Exp Med. (1997) 185:1163–72. doi: 10.1084/jem.185.7.1163 9104803 PMC2196270

[24] Forssmann U Delgado MB Uguccioni M Loetscher P Garotta G Baggiolini M . CKbeta8, a novel CC chemokine that predominantly acts on monocytes. FEBS Lett. (1997) 408:211–6. doi: 10.1016/s0014-5793(97)00408-0 9187369

[25] Berbic M Fraser IS . Immunology of normal and abnormal menstruation. Womens Health (Lond). (2013) 9:387–95. doi: 10.2217/whe.13.32 23826779

[26] Dutta M Joshi M Srivastava S Lodh I Chakravarty B Chaudhury K . A metabonomics approach as a means for identification of potential biomarkers for early diagnosis of endometriosis. Mol Biosyst. (2012) 8:3281–7. doi: 10.1039/c2mb25353d 23079773

[27] Ortiz CN Torres-Reverón A Appleyard CB . Metabolomics in endometriosis: challenges and perspectives for future studies. Reprod Fertil. (2021) 2:R35–50. doi: 10.1530/RAF-20-0047 35128453 PMC8812441

[28] Velingkar A Vuree S Prabhakar PK Kalashikam RR Banerjee A Kondeti S . Fibroblast growth factor 21 as a potential master regulator in metabolic disorders. Am J Physiol Endocrinol Metab. (2023) 324:E409–24. doi: 10.1152/ajpendo.00244.2022 36629821

[29] Fisher FM Maratos-Flier E . Understanding the physiology of FGF21. Annu Rev Physiol. (2016) 78:223–41. doi: 10.1146/annurev-physiol-021115-105339 26654352

[30] Napoli JL . Physiological insights into all-trans-retinoic acid biosynthesis. Biochim Biophys Acta. (2012) 1821:152–67. doi: 10.1016/j.bbalip.2011.05.004 21621639 PMC3179567

[31] Longo N Frigeni M Pasquali M . Carnitine transport and fatty acid oxidation. Biochim Biophys Acta. (2016) 1863:2422–35. doi: 10.1016/j.bbamcr.2016.01.023 26828774 PMC4967041

[32] Matsuzaki S Darcha C . Involvement of the Wnt/β-catenin signaling pathway in the cellular and molecular mechanisms of fibrosis in endometriosis. PloS One. (2013) 8:e76808. doi: 10.1371/journal.pone.0076808 24124596 PMC3790725

[33] Vouk K Hevir N Ribić-Pucelj M Haarpaintner G Scherb H Osredkar J . Discovery of phosphatidylcholines and sphingomyelins as biomarkers for ovarian endometriosis. Hum Reprod. (2012) 27:2955–65. doi: 10.1093/humrep/des152 22859507

