# Supplementary material for: Multi-omics profiling identifies an immunometabolic signature associated with endometriosis
Source: Front Endocrinol (Lausanne). 2026 Aug 11;17:1912463. doi: 10.3389/fendo.2026.1912463 (PMC13503607; doi:10.3389/fendo.2026.1912463)
Supplement: Supplementary file 1 [file DataSheet1.docx]

**Supplementary Material**

**Supplementary Figure S1. Quality control and analytical workflow of Olink inflammatory proteomic profiling**

*(A) Overview of the high-throughput proteomic profiling process based on the Olink target 96 inflammation panel using peripheral blood samples; (B) Boxplot of NPX distribution across all samples. The x-axis represents sample IDs, and the y-axis indicates NPX expression values of proteins. The boxplot includes five elements: minimum (Q1-1.5×IQR, lower whisker), first quartile Q1 (lower edge of the box), median Q2 (black line inside the box), third quartile Q3 (upper edge of the box), and maximum (Q3+1.5×IQR, upper whisker). Black dots beyond the whiskers (above Q3+1.5×IQR or below Q1-1.5×IQR) denote proteins with outlier NPX values; (C) QC_IQR scatterplot showing NPX distribution across all samples. The x-axis shows the mean NPX values for the samples, with two vertical lines indicating mean ± 3 SDs. The y-axis shows the IQR for each sample, with two horizontal lines indicating IQR mean ± 3 SDs; (D) Cluster analysis heatmap. The x-axis represents protein names, and the y-axis represents sample names. Hierarchical clustering was performed on both proteins and samples. Standardized NPX values are color-coded from blue (low) to red (high), with white representing intermediate values.*

**Supplementary Figure S2. Quality Control Assessment of Untargeted Serum Metabolomics Data**

*(A) Principal component analysis (PCA) score plot of quality control (QC) samples and study samples acquired in negative ion mode. QC samples (red) cluster tightly, indicating high analytical stability and minimal instrumental drift during data acquisition. (B) Distribution of relative standard deviation (RSD) values for detected metabolic features in negative ion mode. A total of 70.0% of features exhibited RSD values below 30%, demonstrating acceptable analytical reproducibility. (C) PCA score plot of QC samples and study samples acquired in positive ion mode, showing tight clustering of QC samples and good data consistency throughout the analytical run. (D) Distribution of RSD values for detected metabolic features in positive ion mode. 73.5% of features showed RSD values below 30%, indicating robust analytical performance and reliable metabolomic measurements.*

*Legend: Green dots represent study samples, while red dots represent pooled QC samples. The ellipse denotes the 95% confidence interval in PCA score plots. RSD distributions summarize the reproducibility of detected metabolic features across QC samples, with features exhibiting RSD < 30% considered analytically reliable.*

**Supplementary Figure S3. Distribution of Metabolite Annotation Confidence Levels Based on the Metabolomics Standards Initiative (MSI) Framework**

**Note:** *Numbers above each bar indicate the absolute number of metabolites, while percentages represent their proportion among all annotated metabolites (n = 1,372). MSI, Metabolomics Standards Initiative.*

**Supplementary Figure S4. ROC Validation of EMT-Associated Metabolites**

**Note:** *Bootstrap-validated ROC curves for five differential metabolites distinguishing EMT from Healthy controls. AUC and FDR values are indicated.*

**Supplementary Figure S5. ROC Validation of Benign-Associated Metabolites**

**Note:** *Bootstrap-validated ROC curves for differential metabolites distinguishing Benign from Healthy controls. AUC and FDR values are indicated.*

**Table S1. Mantel test results between microbiome-related immune factors (CCL23, CD8A, CDCP1, FGF21) and metabolomic profiles in patients with endometriosis.**

| **Microbiome** | **Metabolome** | **correlation** | **pvalue** | **Mantels_r** | **Mantels_p** |
| --- | --- | --- | --- | --- | --- |
| CCL23 | Glucoputranjivin | -0.079349738 | 0.957 | < 0.1 | >= 0.05 |
| CCL23 | Aflatoxin Q1 | -0.063562267 | 0.933 | < 0.1 | >= 0.05 |
| CCL23 | 16-Methylheptadecanoic acid | -0.056120994 | 0.866 | < 0.1 | >= 0.05 |
| CCL23 | Androsterone glucuronide | -0.044460717 | 0.728 | < 0.1 | >= 0.05 |
| CCL23 | 2-Oxoadipic acid | -0.036297091 | 0.774 | < 0.1 | >= 0.05 |
| CCL23 | 10beta-hydroxytaxa-4(20),11-dien-5alpha-yl acetate | -0.036279015 | 0.807 | < 0.1 | >= 0.05 |
| CCL23 | CHEMBL441356 | -0.029856116 | 0.734 | < 0.1 | >= 0.05 |
| CCL23 | 4-Hydroxy-6-heneicosanone | -0.026277393 | 0.644 | < 0.1 | >= 0.05 |
| CCL23 | 3-Hydroxy-9Z-octadecenoylcarnitine | -0.021973207 | 0.642 | < 0.1 | >= 0.05 |
| CCL23 | Gingerol | -0.021551906 | 0.54 | < 0.1 | >= 0.05 |
| CCL23 | (2S)-5-(Diaminomethylideneamino)-2-[[2-(2,2-diphenylethoxy)acetyl]amino]pentanoic acid | -0.01971366 | 0.719 | < 0.1 | >= 0.05 |
| CCL23 | Cuminaldehyde | -0.014556023 | 0.581 | < 0.1 | >= 0.05 |
| CCL23 | Biperiden | -0.010657746 | 0.528 | < 0.1 | >= 0.05 |
| CCL23 | Carnitine | -0.008234416 | 0.516 | < 0.1 | >= 0.05 |
| CCL23 | 3-Dehydrocarnitine | -0.008143742 | 0.588 | < 0.1 | >= 0.05 |
| CCL23 | Hexanoylcarnitine | -0.003665495 | 0.527 | < 0.1 | >= 0.05 |
| CCL23 | 5-Hydroxy-1-(3-hydroxy-4-methoxyphenyl)decan-3-one | -0.001912577 | 0.515 | < 0.1 | >= 0.05 |
| CCL23 | 2-Methyl-4-phenyl-2-butyl 2-methylpropanoate | 0.005030735 | 0.402 | < 0.1 | >= 0.05 |
| CCL23 | Butyrylcarnitine | 0.009046602 | 0.401 | < 0.1 | >= 0.05 |
| CCL23 | desmethylastemizole | 0.022442638 | 0.299 | < 0.1 | >= 0.05 |
| CCL23 | 5-Dehydro-4-deoxy-D-glucuronate | 0.025309025 | 0.146 | < 0.1 | >= 0.05 |
| CCL23 | Diacetoxyscirpenol | 0.026435841 | 0.316 | < 0.1 | >= 0.05 |
| CCL23 | 27-Hydroxycholesterol | 0.029722174 | 0.248 | < 0.1 | >= 0.05 |
| CCL23 | 9-cis-Retinoic acid | 0.031985978 | 0.22 | < 0.1 | >= 0.05 |
| CCL23 | 17-Deoxycortolone | 0.033592559 | 0.289 | < 0.1 | >= 0.05 |
| CCL23 | Fluorene | 0.048599161 | 0.168 | < 0.1 | >= 0.05 |
| CCL23 | Gentamicin C2 | 0.06165525 | 0.117 | < 0.1 | >= 0.05 |
| CCL23 | Decanoylcarnitine (C10) | 0.088869111 | 0.095 | < 0.1 | >= 0.05 |
| CCL23 | Tiglylcarnitine | 0.107387195 | 0.027 | 0.1 - 0.2 | 0.01 - 0.05 |
| CCL23 | Acetyl-L-carnitine | 0.14952767 | 0.019 | 0.1 - 0.2 | 0.01 - 0.05 |
| CD8A | Gingerol | -0.088789222 | 0.993 | < 0.1 | >= 0.05 |
| CD8A | Biperiden | -0.086694426 | 0.974 | < 0.1 | >= 0.05 |
| CD8A | 27-Hydroxycholesterol | -0.080326026 | 0.988 | < 0.1 | >= 0.05 |
| CD8A | 4-Hydroxy-6-heneicosanone | -0.068089717 | 0.939 | < 0.1 | >= 0.05 |
| CD8A | 9-cis-Retinoic acid | -0.060092387 | 0.952 | < 0.1 | >= 0.05 |
| CD8A | 17-Deoxycortolone | -0.059559433 | 0.865 | < 0.1 | >= 0.05 |
| CD8A | Aflatoxin Q1 | -0.058617615 | 0.918 | < 0.1 | >= 0.05 |
| CD8A | CHEMBL441356 | -0.057809471 | 0.933 | < 0.1 | >= 0.05 |
| CD8A | Glucoputranjivin | -0.057584201 | 0.85 | < 0.1 | >= 0.05 |
| CD8A | 10beta-hydroxytaxa-4(20),11-dien-5alpha-yl acetate | -0.055429271 | 0.934 | < 0.1 | >= 0.05 |
| CD8A | Gentamicin C2 | -0.055231501 | 0.895 | < 0.1 | >= 0.05 |
| CD8A | 3-Hydroxy-9Z-octadecenoylcarnitine | -0.045359996 | 0.842 | < 0.1 | >= 0.05 |
| CD8A | 16-Methylheptadecanoic acid | -0.042778026 | 0.806 | < 0.1 | >= 0.05 |
| CD8A | Fluorene | -0.033834442 | 0.714 | < 0.1 | >= 0.05 |
| CD8A | Decanoylcarnitine (C10) | -0.026141091 | 0.652 | < 0.1 | >= 0.05 |
| CD8A | Cuminaldehyde | -0.015794397 | 0.583 | < 0.1 | >= 0.05 |
| CD8A | 5-Dehydro-4-deoxy-D-glucuronate | -0.015750056 | 0.712 | < 0.1 | >= 0.05 |
| CD8A | Acetyl-L-carnitine | -0.014220488 | 0.586 | < 0.1 | >= 0.05 |
| CD8A | Butyrylcarnitine | -0.013126929 | 0.6 | < 0.1 | >= 0.05 |
| CD8A | 2-Oxoadipic acid | -0.013030434 | 0.603 | < 0.1 | >= 0.05 |
| CD8A | 3-Dehydrocarnitine | -0.011668833 | 0.633 | < 0.1 | >= 0.05 |
| CD8A | 5-Hydroxy-1-(3-hydroxy-4-methoxyphenyl)decan-3-one | -0.009619516 | 0.586 | < 0.1 | >= 0.05 |
| CD8A | (2S)-5-(Diaminomethylideneamino)-2-[[2-(2,2-diphenylethoxy)acetyl]amino]pentanoic acid | -0.001223897 | 0.475 | < 0.1 | >= 0.05 |
| CD8A | Tiglylcarnitine | 0.001738813 | 0.463 | < 0.1 | >= 0.05 |
| CD8A | 2-Methyl-4-phenyl-2-butyl 2-methylpropanoate | 0.002834118 | 0.415 | < 0.1 | >= 0.05 |
| CD8A | Carnitine | 0.012835979 | 0.366 | < 0.1 | >= 0.05 |
| CD8A | Diacetoxyscirpenol | 0.021285589 | 0.333 | < 0.1 | >= 0.05 |
| CD8A | Hexanoylcarnitine | 0.025585236 | 0.264 | < 0.1 | >= 0.05 |
| CD8A | Androsterone glucuronide | 0.058881097 | 0.147 | < 0.1 | >= 0.05 |
| CD8A | desmethylastemizole | 0.078627887 | 0.087 | < 0.1 | >= 0.05 |
| CDCP1 | CHEMBL441356 | -0.084771429 | 0.959 | < 0.1 | >= 0.05 |
| CDCP1 | Gentamicin C2 | -0.0621168 | 0.834 | < 0.1 | >= 0.05 |
| CDCP1 | 27-Hydroxycholesterol | -0.055403005 | 0.87 | < 0.1 | >= 0.05 |
| CDCP1 | Androsterone glucuronide | -0.053027714 | 0.785 | < 0.1 | >= 0.05 |
| CDCP1 | Aflatoxin Q1 | -0.050986218 | 0.822 | < 0.1 | >= 0.05 |
| CDCP1 | Diacetoxyscirpenol | -0.049076533 | 0.687 | < 0.1 | >= 0.05 |
| CDCP1 | Glucoputranjivin | -0.046466455 | 0.709 | < 0.1 | >= 0.05 |
| CDCP1 | 3-Hydroxy-9Z-octadecenoylcarnitine | -0.038919685 | 0.739 | < 0.1 | >= 0.05 |
| CDCP1 | 2-Oxoadipic acid | -0.036823547 | 0.717 | < 0.1 | >= 0.05 |
| CDCP1 | 3-Dehydrocarnitine | -0.035958161 | 0.837 | < 0.1 | >= 0.05 |
| CDCP1 | desmethylastemizole | -0.033142037 | 0.648 | < 0.1 | >= 0.05 |
| CDCP1 | Gingerol | -0.032936077 | 0.601 | < 0.1 | >= 0.05 |
| CDCP1 | 17-Deoxycortolone | -0.018864536 | 0.545 | < 0.1 | >= 0.05 |
| CDCP1 | Butyrylcarnitine | -0.016589952 | 0.601 | < 0.1 | >= 0.05 |
| CDCP1 | 9-cis-Retinoic acid | -0.015547337 | 0.57 | < 0.1 | >= 0.05 |
| CDCP1 | Fluorene | -0.010194838 | 0.487 | < 0.1 | >= 0.05 |
| CDCP1 | 5-Dehydro-4-deoxy-D-glucuronate | -0.001072957 | 0.479 | < 0.1 | >= 0.05 |
| CDCP1 | Acetyl-L-carnitine | 0.000768594 | 0.445 | < 0.1 | >= 0.05 |
| CDCP1 | Carnitine | 0.01766188 | 0.339 | < 0.1 | >= 0.05 |
| CDCP1 | Cuminaldehyde | 0.019497554 | 0.331 | < 0.1 | >= 0.05 |
| CDCP1 | Hexanoylcarnitine | 0.020179087 | 0.355 | < 0.1 | >= 0.05 |
| CDCP1 | 16-Methylheptadecanoic acid | 0.025348903 | 0.31 | < 0.1 | >= 0.05 |
| CDCP1 | Biperiden | 0.031950326 | 0.267 | < 0.1 | >= 0.05 |
| CDCP1 | (2S)-5-(Diaminomethylideneamino)-2-[[2-(2,2-diphenylethoxy)acetyl]amino]pentanoic acid | 0.03216144 | 0.181 | < 0.1 | >= 0.05 |
| CDCP1 | 10beta-hydroxytaxa-4(20),11-dien-5alpha-yl acetate | 0.036638598 | 0.203 | < 0.1 | >= 0.05 |
| CDCP1 | Decanoylcarnitine (C10) | 0.03820364 | 0.275 | < 0.1 | >= 0.05 |
| CDCP1 | 4-Hydroxy-6-heneicosanone | 0.053475568 | 0.185 | < 0.1 | >= 0.05 |
| CDCP1 | 2-Methyl-4-phenyl-2-butyl 2-methylpropanoate | 0.055733566 | 0.164 | < 0.1 | >= 0.05 |
| CDCP1 | Tiglylcarnitine | 0.061543171 | 0.142 | < 0.1 | >= 0.05 |
| CDCP1 | 5-Hydroxy-1-(3-hydroxy-4-methoxyphenyl)decan-3-one | 0.077996948 | 0.034 | < 0.1 | 0.01 - 0.05 |
| FGF21 | Carnitine | -0.064338765 | 0.957 | < 0.1 | >= 0.05 |
| FGF21 | Diacetoxyscirpenol | -0.054818454 | 0.846 | < 0.1 | >= 0.05 |
| FGF21 | Acetyl-L-carnitine | -0.051263207 | 0.819 | < 0.1 | >= 0.05 |
| FGF21 | Butyrylcarnitine | -0.040656757 | 0.826 | < 0.1 | >= 0.05 |
| FGF21 | Hexanoylcarnitine | -0.031608087 | 0.758 | < 0.1 | >= 0.05 |
| FGF21 | CHEMBL441356 | -0.031593091 | 0.76 | < 0.1 | >= 0.05 |
| FGF21 | 3-Dehydrocarnitine | -0.013319388 | 0.648 | < 0.1 | >= 0.05 |
| FGF21 | Cuminaldehyde | -0.010876859 | 0.545 | < 0.1 | >= 0.05 |
| FGF21 | (2S)-5-(Diaminomethylideneamino)-2-[[2-(2,2-diphenylethoxy)acetyl]amino]pentanoic acid | -0.008041209 | 0.585 | < 0.1 | >= 0.05 |
| FGF21 | Androsterone glucuronide | -0.005799399 | 0.452 | < 0.1 | >= 0.05 |
| FGF21 | 5-Hydroxy-1-(3-hydroxy-4-methoxyphenyl)decan-3-one | -0.003898868 | 0.489 | < 0.1 | >= 0.05 |
| FGF21 | 5-Dehydro-4-deoxy-D-glucuronate | -0.003381132 | 0.547 | < 0.1 | >= 0.05 |
| FGF21 | 2-Oxoadipic acid | -0.00056319 | 0.476 | < 0.1 | >= 0.05 |
| FGF21 | Decanoylcarnitine (C10) | 0.001770162 | 0.447 | < 0.1 | >= 0.05 |
| FGF21 | 27-Hydroxycholesterol | 0.008095407 | 0.379 | < 0.1 | >= 0.05 |
| FGF21 | 17-Deoxycortolone | 0.020754923 | 0.329 | < 0.1 | >= 0.05 |
| FGF21 | 10beta-hydroxytaxa-4(20),11-dien-5alpha-yl acetate | 0.020882754 | 0.285 | < 0.1 | >= 0.05 |
| FGF21 | 16-Methylheptadecanoic acid | 0.021636388 | 0.276 | < 0.1 | >= 0.05 |
| FGF21 | Aflatoxin Q1 | 0.026773233 | 0.266 | < 0.1 | >= 0.05 |
| FGF21 | 4-Hydroxy-6-heneicosanone | 0.029341952 | 0.259 | < 0.1 | >= 0.05 |
| FGF21 | Gentamicin C2 | 0.038964429 | 0.207 | < 0.1 | >= 0.05 |
| FGF21 | Glucoputranjivin | 0.04332304 | 0.214 | < 0.1 | >= 0.05 |
| FGF21 | 2-Methyl-4-phenyl-2-butyl 2-methylpropanoate | 0.048774886 | 0.148 | < 0.1 | >= 0.05 |
| FGF21 | Gingerol | 0.052446214 | 0.137 | < 0.1 | >= 0.05 |
| FGF21 | Tiglylcarnitine | 0.061722698 | 0.088 | < 0.1 | >= 0.05 |
| FGF21 | 9-cis-Retinoic acid | 0.066799002 | 0.058 | < 0.1 | >= 0.05 |
| FGF21 | 3-Hydroxy-9Z-octadecenoylcarnitine | 0.068702818 | 0.08 | < 0.1 | >= 0.05 |
| FGF21 | Biperiden | 0.074446212 | 0.11 | < 0.1 | >= 0.05 |
| FGF21 | desmethylastemizole | 0.087397552 | 0.063 | < 0.1 | >= 0.05 |
| FGF21 | Fluorene | 0.142603194 | 0.012 | 0.1 - 0.2 | 0.01 - 0.05 |

**Supplementary Figure S6. Group-Adjusted Protein–Metabolite Correlation Network**

**Note:** *Heatmap showing significant protein–metabolite correlations after adjustment for diagnostic group effects. Correlation coefficients are represented by color intensity, with red indicating positive correlations and blue indicating negative correlations. Only associations remaining significant after FDR correction are displayed. AXIN1, SIRT2, and IL8 exhibited the strongest metabolite associations, highlighting persistent immune–metabolic interactions independent of group-level differences.*

**Supplementary Figure S7. Cross-Validated Performance of Protein, Metabolite, and Integrated Multi-Omics Models**

**Note:** *Mean cross-validated AUC values (± SD) obtained from repeated stratified five-fold cross-validation for protein-only, metabolite-only, and combined protein–metabolite models across the three group comparisons. Metabolite-based and integrated multi-omics models consistently outperformed protein-only models, with the highest performance observed for the combined model in the Benign versus Healthy comparison. Error bars indicate standard deviations across validation iterations.*
